# BH3 mimetics augment cytotoxic T cell killing of acute myeloid leukemia via mitochondrial apoptotic mechanism

**DOI:** 10.1038/s41420-025-02375-2

**Published:** 2025-03-26

**Authors:** Kapil Saxena, Shao-Hsi Hung, Esther Ryu, Shailbala Singh, Qi Zhang Tatarata, Zhihong Zeng, Zhe Wang, Marina Y. Konopleva, Cassian Yee

**Affiliations:** 1 Division of Cancer Medicine, The University of Texas MD Anderson Cancer Center, Houston, TX USA; 2 Department of Melanoma Medical Oncology, The University of Texas MD Anderson Cancer Center, Houston, TX USA; 3 The University of Texas MD Anderson Cancer Center UTHealth Houston Graduate School of Biomedical Sciences, Houston, TX USA; 4 Department of Leukemia, The University of Texas MD Anderson Cancer Center, Houston, TX USA; 5 Department of Immunology, The University of Texas MD Anderson Cancer Center, Houston, TX USA; 6 Present Address: Gilead Sciences Inc., Foster City, CA USA; 7 Present Address: Department of Medicine, State University of New York Downstate Health Sciences University, Brooklyn, NY USA; 8 Present Address: Division of Immunobiology, Cincinnati Children’s Hospital Medical Center, Cincinnati, OH USA; 9 Present Address: Department of Oncology and Molecular Pharmacology, Albert Einstein College of Medicine, Bronx, NY USA

**Keywords:** Acute myeloid leukaemia, Acute myeloid leukaemia, Apoptosis

## Abstract

Adoptive cell therapy (ACT) can address an unmet clinical need for patients with relapsed/refractory acute myeloid leukemia (AML), but its effect is often modest in the setting of high tumor burden. In this study, we postulated that strategies to lower the AML apoptotic threshold will augment T cell killing of AML cells. BH3 mimetics, such as venetoclax, are a clinically approved class of compounds that predispose cells to intrinsic apoptosis by inhibiting anti-apoptotic mitochondrial proteins. We explored the anti-leukemic efficacy of BH3 mimetics combined with WT1-specific CD8+ T cells on AML cell lines and primary samples from patients with a diverse array of disease characteristics to evaluate if lowering the cellular apoptotic threshold via inhibition of anti-apoptotic mitochondrial proteins can increase leukemic cell sensitivity to T cell therapy. We found that the combination approach of BH3 mimetic and CD8+ T cells led to significantly increased killing of established AML lines as well as of adverse-risk primary AML leukemic blast cells. In contrast to the hypothesis that enhanced killing would be due to combined activation of the intrinsic and extrinsic apoptotic pathways, our data suggests that CTL-mediated killing of AML cells was accomplished primarily through activation of the intrinsic/mitochondrial apoptotic pathway. This highly effective combinatorial activity due to convergence on the mitochondrial apoptotic pathway was conserved across multiple AML cell lines and primary samples, suggesting that mitochondrial priming may represent a novel mechanism of optimizing adoptive cell therapy for AML patients.

## Introduction

The development of chimeric antigen receptor (CAR) T cell therapy as standard of care for several B-cell malignancies has raised the prospect of similar cell therapy-based approaches for cancers such as acute myeloid leukemia (AML) [1, 2]. For many patients with AML, allogeneic stem cell transplantation (ASCT) remains the consolidative approach with the highest likelihood of achieving long-term disease control [3, 4]. Despite improvements in conditioning regimens, supportive care, donor availability, and graft-versus-host disease (GVHD) management, most AML patients do not undergo ASCT due to not having sufficient disease response to induction therapy or pre-existing comorbidities that preclude the intensive treatment approach of ASCT, with only a minority of otherwise eligible patients not proceeding to ASCT due to favorable disease characteristics [3, 5, 6]. Furthermore, for those who do receive ASCT, relapse rates can be as high as 50% for patients with adverse-risk disease characteristics [7].

Due to the above challenges of ASCT, other forms of adoptive cell therapy are currently under investigation for patients with AML. CAR-T cells have been tested for AML in clinical trials, with targets such as CD33, CD38, CLL-1, and CD123 primarily under investigation [2]. Though some of these targets have shown clinical responses in a subset of patients, the approach largely remains experimental [2, 8, 9]. No CAR-T product for AML has received formal approval, and concerns remain about on-target off-tumor effects on normal hematopoietic cells because none of these surface targets are exclusively expressed by leukemic cells.

In contrast to CAR-T therapy, T cell receptor (TCR) based strategies for AML can target both surface and intracellular proteins because the TCR recognizes a peptide derived from antigen in any cellular compartment, which is then presented by a major histocompatibility complex (MHC). For CD8+ cytotoxic T lymphocytes (CTLs), these peptide/MHC (pMHC) targets typically consist of an 8–11 amino acid peptide chain presented on a human lymphocyte antigen (HLA) molecule [10]. WT1, a prototypic TCR target, is the most common target for clinical trials in AML, with both engineered TCR-T cell and non-engineered endogenous T cell (ETC) approaches being employed [11–13]. ETC therapy targeting a WT1 peptide uses non-transduced, non-engineered WT1-specific CTLs that have been primed and expanded in vitro from rare populations of peripheral blood-derived WT1-specific T cells and epigenetically programmed into central memory T cells (Tcm), which can elicit long-lasting immune protection against relapse in patients with high-risk AML [12].

However, as observed with CAR-T and bispecific antibody therapy in B-cell malignancies, CTLs have shown reduced efficacy in patients with high AML leukemic burden, perhaps due to reduced T cell cytotoxicity in vivo even when immunogenic targets have been identified [13–16]. In order to achieve more effective anti-leukemic therapy, we postulate that a CTL combination partner that can shift the effective CTL to AML cell equation, either by improving CTL cytotoxicity directly, reducing AML tumor burden, or lowering the apoptotic threshold of AML cells, may improve adoptive cell therapy for AML. Thus far, no small molecule has been approved in combination with an adoptive cellular product, and many studies largely focus on the use of additional immune-active compounds (such as immune checkpoint inhibitors) [17]. However, specific classes of small molecules are already in oncologic clinical use that may improve the efficacy of adoptive cellular therapies by augmenting T cell cytotoxicity. Because CTLs primarily cause tumor cell death via the induction of apoptosis, we chose to explore whether non-overlapping modalities using BH3 mimetics (activators of intrinsic apoptosis through inhibition of BCL-2 family anti-apoptotic proteins) and CTLs (primarily extrinsic apoptosis activators) can lead to enhanced anti-leukemic activity through apoptotic pathway crosstalk in which both the intrinsic and extrinsic apoptotic pathways are activated in the same tumor cell [18–20]. Notably, although CTL-mediated killing is commonly attributed to activating extrinsic apoptosis, it has been shown that granzyme B can lead to activation of caspase 9, which is involved in intrinsic apoptosis, as well as directly cleave caspase 3 and the pro-apoptotic protein Bid [21–23]. Though the commonly implicated inducers of intrinsic apoptosis are DNA damage, hypoxia, oxidative stress, and withdrawal of growth factors/nutrients, the potential of granzyme B to also activate intrinsic apoptosis makes this apoptotic pathway a potentially underappreciated form of CTL-induced cytotoxicity [21, 24]. This may be particularly relevant in CTL-based combination therapies given the concept of the “apoptotic cliff” for intrinsic/mitochondrial apoptosis, in which multiple intrinsic apoptotic stimuli can ultimately commit a cell to irreversible mitochondrial outer membrane permeabilization (MOMP) [25].

Venetoclax (VEN) is a BCL-2 inhibitor and the only BH3 mimetic which is currently approved for clinical use in any type of malignancy [26]. VEN has been shown to work in both additive and synergistic means to enhance AML cell death by sensitizing AML cells to subsequent apoptotic insults (such as chemotherapeutic agents or small molecule inhibitors), and it is FDA approved for use in AML in combination with low-intensity therapies [27, 28]. To date, VEN has not been approved for use with immune-based therapies in any type of malignancy. In contrast to most chemotherapeutic agents, VEN has minimal toxicity against nonhematopoietic cells, its hematologic toxicities are largely restricted to reversible cytopenias, and preclinical data suggests that VEN has limited toxicity on T cells [29–31]. VEN has been studied in preclinical models in combination with NK cells, CAR-T cells, and double-negative T cells (CD4−/CD8−; DNTs) [29, 32, 33]. CD8+ CTLs are the most common cytotoxic effector cell in circulation, and knowledge of how to improve CD8+ CTL efficacy in AML with potential partners such as BH3 mimetics remains an area of clinical importance.

In the current study, we evaluated if BH3 mimetics such as VEN can be used in combination with WT1 pMHC-specific CD8+ CTLs to augment AML cell killing through apoptotic pathway crosstalk. Utilizing multiple AML cell lines, we demonstrate that the benefit of VEN in combination with CTLs is largely mediated by activation of apoptosis in VEN-sensitive AML cells; there was minimal direct enhancement of T cell cytotoxicity by VEN, contrary to a prior report in DNTs [29]. Notably, we found that this combination efficacy was not due to apoptotic pathway crosstalk and rather due to T cell-mediated induction of the mitochondrial apoptotic pathway in AML cells, which was further enhanced by combination with VEN. Furthermore, we demonstrate that combination cytotoxic activity was reproducible in primary AML samples from multiple patients with a diverse array of mutations, cytogenetic changes, and prior treatments. We show that a pretreatment approach of VEN followed by CTLs minimizes toxicity of VEN on CTLs while still leading to enhanced AML cell killing, including of the CD34+/CD38− compartment, compared to either therapy alone. Lastly, we demonstrate that in AML cells with a predisposition to developing VEN resistance based on their mutational profile, substitution of VEN with an MCL-1 inhibitor can be used in combination with CTLs to augment AML cell death.

## Results

### WT1-specific CTLs are cytotoxic against AML cells

**Fig. 1:**
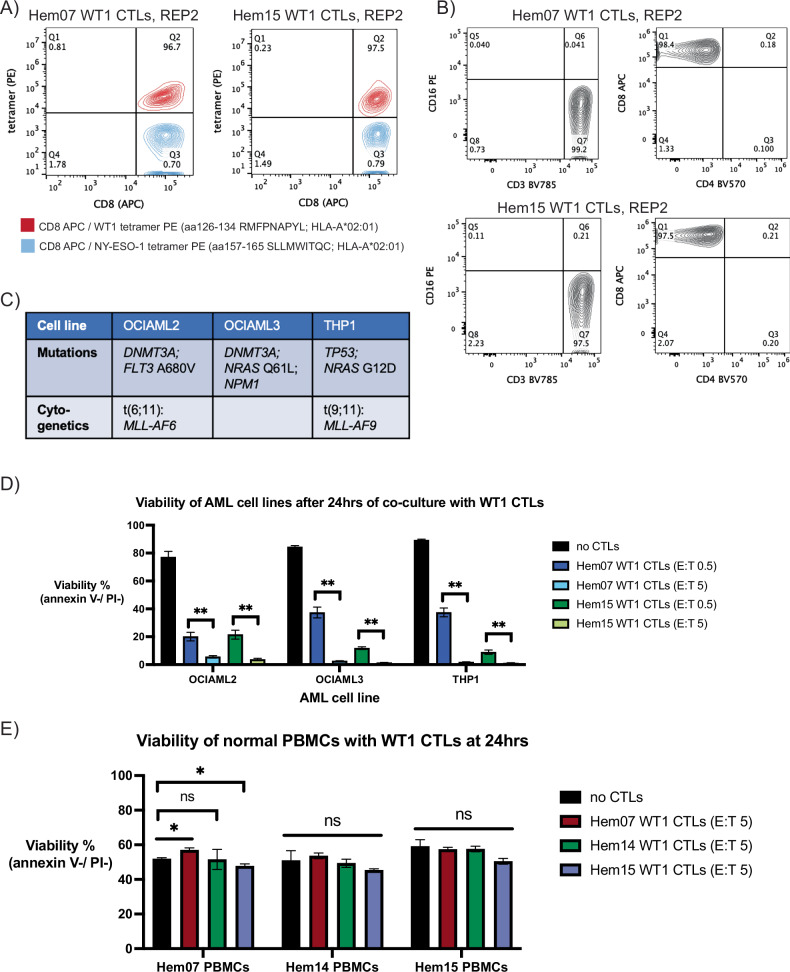
Characterization of ETC-protocol generated WT1-CTLs. **A** Tetramer staining with a control tetramer (NY-ESO-1, blue) and a WT1 tetramer (red) on ETC-generated CTLs from donors Hem07 and Hem15. **B** Surface staining on ETC-generated WT1-CTLs from donors Hem07 and Hem15 for CD3 vs CD16 and CD4 vs CD8; gating performed using matched PBMCs from each donor to differentiate positive vs negative populations (PBMC data not shown). **C** Notable mutational and cytogenetic changes in the 3 HLA-A*02:01 + AML cell lines. **D** AML cell viability after 24 h of co-culture with WT1-CTLs from donors Hem07 and Hem15. Viability was assessed at 24 h with staining on unfixed cells for annexin V (FITC) and propidium iodide (PI). Viable cells were quantified as the double-negative population. Gating strategy shown in Supplementary Fig. 1F. Black bar shows the baseline viability of the AML cells at 24 h if no CTLs were added. Asterisks (**) indicate *p* < 0.01 using an unpaired *t*-test between E:T 0.5 and E:T 5 for the respective AML cell line/CTL donor combination. **E** Viability of normal donor PBMCs after 24 h of co-culture with WT1-CTLs from Hem07, Hem14, and Hem15 donors at E:T 5. Baseline viability of PBMCs without CTL co-culture shown in black. Statistical analyses were performed as a one-way ANOVA within each PBMC group between the 4 treatment conditions (no CTLs, Hem07 WT1-CTLs, Hem14 WT1-CTLs, and Hem15 WT1-CTLs) followed by post-hoc testing with an unpaired *t*-test with a Bonferroni adjustment incorporated for significance (*p*/n) where n equals the number of paired comparisons per group (3 comparisons per group), yielding *p < 0.05/3 (*p* < 0.017). Results in (**D**) and (**E**) are shown as mean ± standard deviation of *n* = 4 (**D**) or *n* = 3 (**E**) samples and are representative of 4 (**D**) or 2 (**E**) independent experiments. E:T = effector:target ratio.

For experiments assessing pMHC-specific CTLs on AML cells, we generated CTLs targeting the HLA-A*02:01-restricted 9 amino acid peptide RMFPNAPYL from the protein WT1. WT1 was chosen as a target as it is overexpressed in blasts from >70% of AML patients and present at low levels in normal tissue (such as kidney podocytes and hematopoietic cells) [34]. Though WT1 can be mutated in 6–15% of AML cases, the region encoding the described 9mer (amino acids 126–134) is not a commonly mutated region [35]. Furthermore, CTLs targeting this WT1 9mer (RMFPNAPYL) on an HLA-A*02:01 molecule are one of the most common CTL types studied clinically in AML patients and have been safely employed in multiple AML clinical trials, with rare instances of graft-versus-host toxicities or on-target off-tumor side effects [11–13]. Utilizing a previously described ETC protocol with IL-21 priming (non-transduced, non-engineered T cells), we generated CD8+ T cell populations that were >95% tetramer-positive against the WT1 9mer peptide from multiple healthy donors (Fig. 1A, Supplementary Fig. 1A) with a uniform CD3+/CD4−/CD8+/CD16- immunophenotype (Fig. 1B, Supplementary Fig. 1B) [36]. In vitro, ETC-generated WT1-CTLs primarily display a Teff/Tem phenotype (CCR7-/CD45RA-) (Supplementary Fig. 1B), although a population of Tcm cells consistently emerges in vivo in patients when IL-21 is used during ETC generation [12]. The ETC-generated CTLs displayed dose-dependent cytotoxicity on WT1 peptide-pulsed T2 cells, demonstrating CTL specificity for the pMHC target with affinity between 1 and 10 ng/ml of the target WT1 peptide, and WT1-CTLs did not kill unpulsed T2 cells (Supplementary Fig. 1C). In addition, unselected CD8+ T cells that had not undergone the ETC generation process (which entails sorting for WT1-specific clones and subsequent expansion) did not display notable AML cell killing (Supplementary Fig. 1D).

Next, WT1/HLA-A2-specific CTL (hereon abbreviated as WT1-CTL) cytotoxicity was assessed against a panel of HLA-A*02:01-positive AML cell lines: OCI-AML2, OCI-AML3, and THP-1. These 3 cell lines were chosen due to their HLA-A2+ status as well as diversity of mutational/cytogenetic abnormalities (Fig. 1C). WT1-CTLs from all 3 donors tested displayed significant cytotoxicity against the 3 AML cell lines at both low and high effector:target (E:T) ratios, with <50% of AML cells remaining viable at an E:T of 0.5 and <10% alive at an E:T of 5 (Fig. 1D, Supplementary Fig. 1E, F). In addition, the WT1-CTLs did not demonstrate significant cytotoxicity against either allogeneic or autologous normal donor PBMCs at an E:T ratio of 5, suggesting that killing of the AML cell lines is not mediated merely by HLA-mismatch or a mixed lymphocyte reaction (Fig. 1E).

### Venetoclax and WT1-CTLs can independently and concurrently kill AML cells

**Fig. 2:**
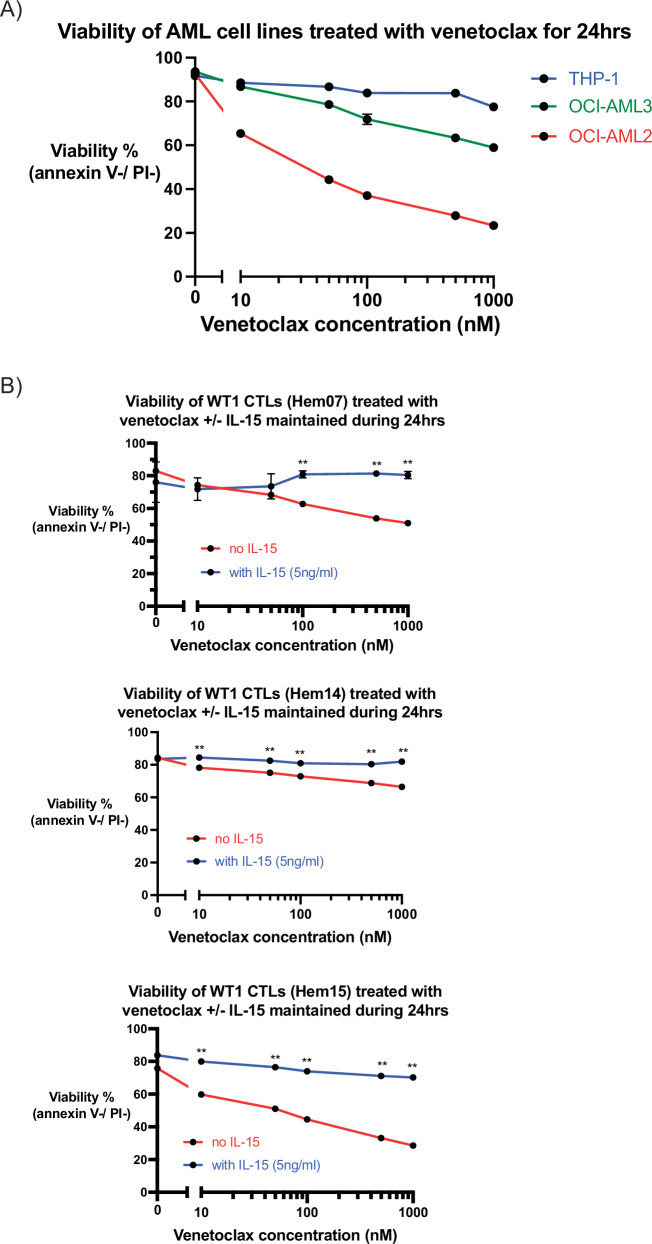
Effect of venetoclax on AML cell lines and WT1-CTLs. **A** AML cell lines were incubated with venetoclax for 24 h in A221 media at the indicated concentrations (0, 10 nM, 50 nM, 100 nM, 500 nM, 1000 nM). Viability was assessed at 24 h using annexin V/PI staining. **B** WT1-CTLs from donors Hem07 (top), Hem14 (middle), or Hem15 (bottom) were first rested for 24 h in CTL media with IL-15 (5 ng/ml). After 24 h of resting, CTLs were transferred to CTL media without IL-15 (red lines) or with IL-15 5 ng/ml (blue lines) and with varying concentrations of venetoclax (0, 10 nM, 50 nM, 100 nM, 500 nM, 1000 nM). Viability was assessed after 24 h of venetoclax exposure using annexin V/PI staining. Statistical analyses were performed as a two-way ANOVA followed by post-hoc *t*-test with Bonferroni adjustment for multiple comparisons; ** = adjusted *p* < 0.01. Results are shown as mean ± standard deviation of *n* = 4 samples and are representative of 3 (**A**) or 2 (**B**) independent experiments.

Prior to testing VEN in conjunction with CTLs, we first evaluated the toxicity of VEN in isolation on AML cell lines and CTLs. Approximately 70–80% of AML patients are sensitive to VEN in the frontline setting, with VEN-resistant patients often having mutations in *TP53* or *RAS*-pathway genes [32, 37–39]. The half maximal inhibitory concentration (IC50) after 24 h of VEN exposure was 36 nM for OCI-AML2 cells [95% confidence interval (CI) 27.8 nM–45.3 nM] (Fig. 2A). The IC50 was not reached for THP-1 (*TP53*- and *RAS*-mutated) nor OCI-AML3 (*RAS*-mutated) cells when testing VEN doses up to 1000 nM (Fig. 2A). It is notable that WT1-CTLs were able to kill the VEN-resistant AML cell lines (Fig. 1D), demonstrating that CTLs can clear VEN-resistant clones. Next, CTLs were treated with VEN to assess for toxicity. The reliance of T cells on various anti-apoptotic BCL-2 family members (such as BCL-2, MCL-1, BCL-XL) varies in studies dependent on (1) mouse versus human models, (2) naïve versus memory status, and (3) inactivated versus activated status [32, 40, 41]. For human ETC therapies, tetramer-specific CTLs are isolated from PBMCs, expanded in the presence of feeder cells + anti-CD3 (OKT-3) + low-dose IL-2, and cryopreserved for subsequent use [42, 43]. For the current in vitro experiments, WT1-CTLs were thawed and rested overnight in low-dose IL-15 (5 ng/ml). Under these conditions, we found that CTLs from 3 different donors survived VEN treatment, although with dose-dependent VEN cytotoxicity that varied by donor (Fig. 2B). However, if CTLs were maintained in low-dose IL-15 (5 ng/ml) during VEN exposure as well, the cytotoxic effects of VEN were largely abrogated, which is compatible with prior reports that IL-15 can regulate certain members of the BCL-2 family of proteins (Fig. 2B) [44, 45].

**Fig. 3:**
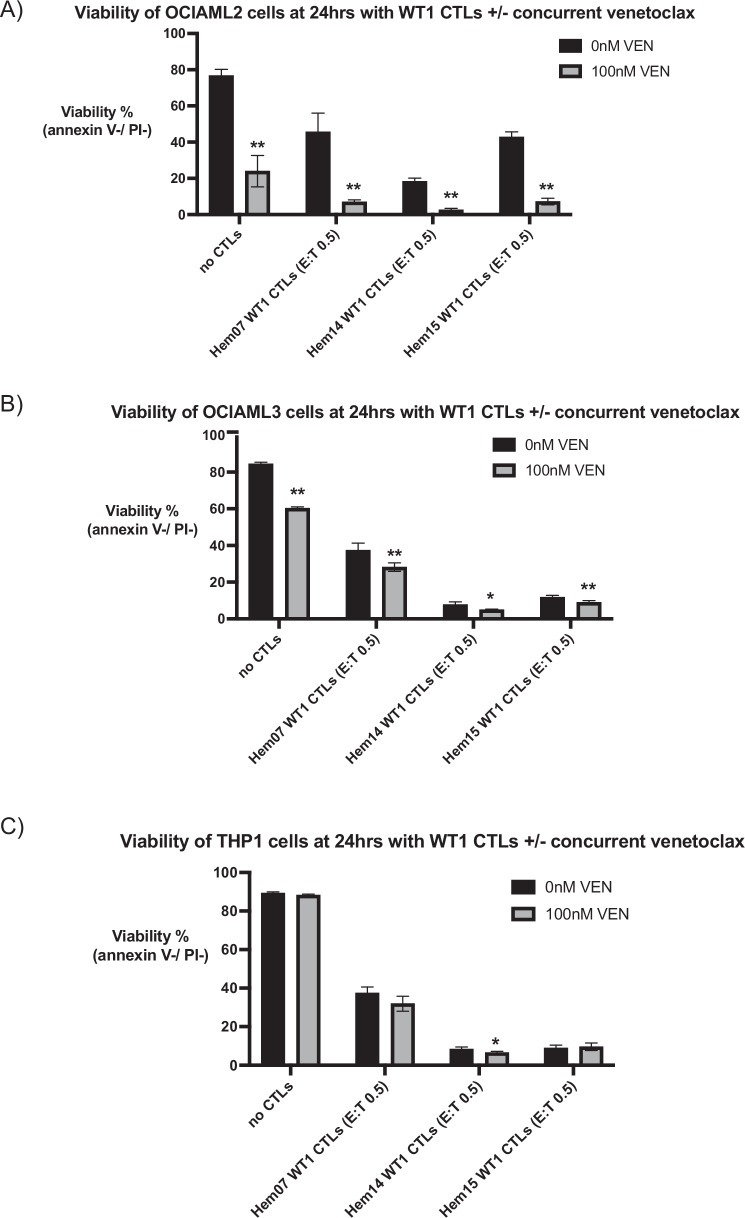
Co-treatment of VEN + CTLs on AML cell lines. AML cell lines OCI-AML2 (**A**), OCI-AML3 (**B**), and THP-1 (**C**) were co-cultured with WT1-CTLs (E:T 0.5) ± venetoclax 100 nM for 24 h. Viability was assessed at 24 h using annexin V/PI staining. Results are shown as mean ± standard deviation of *n* = 4 samples and are representative of 3 independent experiments with 3 different ETC-generated donor-derived WT1-CTLs. Asterisks are comparisons of each pair of black vs gray bars. *p < 0.05; **p < 0.01 using unpaired *t*-test.

A significant challenge with concurrent treatment of patients with cellular therapies and chemotherapeutic agents or small molecule inhibitors is that these agents/inhibitors are often cytotoxic to the cellular therapies themselves, thus obviating simultaneous therapy. As demonstrated in Fig. 2, VEN had toxicity against the CTLs. However, up to doses of 100 nM, >50% of the starting population of CTLs remained alive from all 3 donors; thus, we chose 100 nM for co-treatment assays between VEN and WT1-CTLs. We concurrently treated the 3 previously described AML cell lines with VEN ± WT1-CTLs, thereby aiming to potentially activate both the intrinsic apoptotic pathway (VEN) and the extrinsic apoptotic pathway (CTLs) simultaneously. Utilizing a low E:T ratio (0.5) and VEN at 100 nM, we found that cytotoxicity was increased with the combination of CTLs + VEN on OCI-AML2 cells but not on VEN-resistant THP-1 cell lines and only minimally on OCI-AML3 cells, suggesting that the efficacy of combination therapy is based on the AML cell’s baseline sensitivity to VEN (Fig. 3A–C). When treated with Hem07 WT1-CTLs (E:T 0.5), 45.6% of OCI-AML2 cells were alive at 24 h; co-treatment with VEN 100 nM dropped AML cell viability to an average of 6.9%. Similar results were seen with Hem14 and Hem15 WT1-CTLs (Fig. 3A). As PBMCs were resistant to WT1-CTLs (Fig. 1E), we also assessed the effect of VEN + CTLs on PBMCs and found that VEN + CTLs was not more toxic to PBMCs compared to VEN alone; VEN did not sensitize PBMCs to CTL-mediated killing (Supplementary Fig. 2A). In order to assess if the effects of VEN during CTL co-culture on AML cells was due to an effect on TCR/MHC regulation, we evaluated MHC expression by AML cells and TCR expression by CTLs following VEN exposure. We found that VEN (100 nM) did not increase HLA-A2 or MHC class I expression on any of the 3 AML cell lines nor did it increase TCRα/β expression by any of the 3 CTL donors (Supplementary Fig. 2B, C).

Lastly, given the concerns of direct VEN toxicity on resting CTLs observed in Fig. 2B, we also assessed if CTL viability was affected when CTLs were in co-culture with AML cells with or without VEN (100 nM). We observed a drop in CTL viability with VEN during co-culture with OCI-AML2 cells but not with OCI-AML3 cells or THP-1 cells (Supplementary Fig. 2D). It is notable that co-culture between the VEN-sensitive OCI-AML2 cell line and CTLs + VEN was the one scenario (Fig. 3A) in which AML cell death was significantly superior to CTL treatment alone, suggesting that the additional CTL viability drop seen during OCI-AML2 co-culture (as opposed to co-culture with the VEN-resistant OCI-AML3 nor THP-1 lines) may be due to increased OCI-AML2 cell death and/or increased CTL killing.

### Venetoclax can pre-sensitize AML cells to WT1-CTL-mediated cytotoxicity

**Fig. 4:**
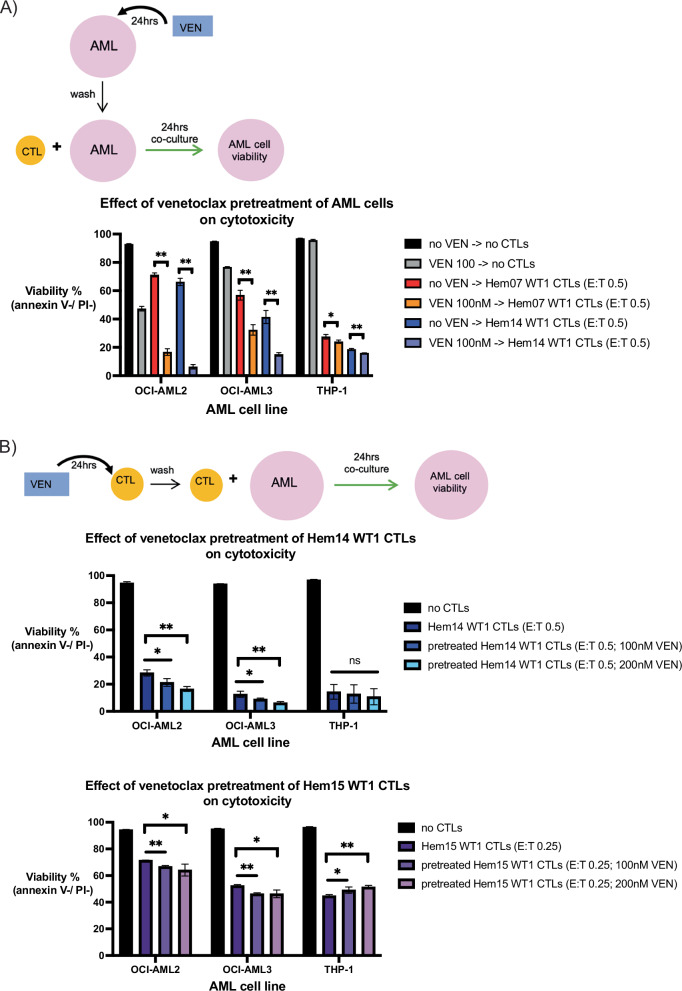
Pre-treatment studies of VEN on AML cells or VEN on CTLs. **A** AML cell lines were either pretreated with or without VEN 100 nM in A221 media for 24 h. After VEN exposure, AML cells were washed three times to remove VEN and then were placed in culture with or without WT1-CTLs (E:T 0.5) for an additional 24 h. At the conclusion of 24 h of co-culture (48 h from experiment initiation), AML cell viability was assessed by annexin V/PI staining. Asterisks are for comparisons of the indicated CTL treatment alone vs VEN− > CTL treatment; *p < 0.05; **p < 0.01 using unpaired *t*-test. **B** WT1-CTLs were either pretreated with or without VEN (100 nM and 200 nM) in CTL media containing IL-15 (5 ng/ml) for 24 h. After VEN exposure, CTLs were washed three times and then placed in co-culture with AML cells (E:T 0.5 for Hem14 CTLs on top row, E:T 0.25 for Hem15 CTLs on bottom row) for an additional 24 h. At the conclusion of 24 h of co-culture (48 h from experiment initiation), AML cell viability was assessed by annexin V/PI staining. Asterisks are for comparisons of co-culture with untreated CTLs vs VEN-treated CTLs. Statistical analyses were performed as a one-way ANOVA within each AML cell line between the 3 CTL treatment conditions (CTLs, VEN 100 nM-treated CTLs, and VEN 200nM-treated CTLs) followed by post-hoc testing with an unpaired *t*-test with a Bonferroni adjustment incorporated for significance (*p*/n) where n equals the number of paired comparisons per group (2 comparisons per group), yielding *p < 0.05/2 (*p* < 0.025) and **p < 0.01/2 (*p* < 0.005). Results are shown as mean ± standard deviation of *n* = 4 samples and are representative of 3 (**A**) or 2 (**B**) independent experiments. Schematics of the experimental setup are shown above each figure.

It has previously been shown that VEN may act on a relatively rare subset of T cells (double-negative CD4−/CD8−T cells; DNTs) to directly increase their cytotoxic potential [29]. Based on these data, we performed pretreatment studies to assess if the increased AML cell death seen with CTLs + VEN on OCI-AML2 cells (and to some extent on OCI-AML3 cells) was due to a similar phenomenon of direct activity by VEN on the CTLs to increase their killing capacity or was due to a combined effect by both VEN and CTLs together on the AML cells. First, we tested pretreatment with VEN on AML cells (with VEN subsequently washed off) followed by WT1-CTL co-culture, modeling a clinical scenario in which VEN is given to a patient followed by CTL infusion 24 h afterward. Under this scenario, we found that AML pretreatment with VEN followed by WT1-CTL co-culture led to increased AML cell death in OCI-AML2 and OCI-AML3 cells with additive efficacy (Fig. 4A, Supplementary Fig. 3A). With no CTL nor VEN treatment for 48 h, OCI-AML2 viability averaged 92.9%. After VEN 100 nM for 24 h (followed by 24 h in media alone without VEN or CTLs), this viability dropped to 47.3%. With WT1-CTL treatment (media for 24 h and then CTLs for 24 h), viability dropped to 71.2% with Hem07 WT1-CTLs (E:T 0.5) and 66.2% with Hem14 WT1-CTLs (E:T 0.5). However, when OCI-AML2 cells were treated with VEN first for 24 h and then WT1-CTLs for 24 h, viability dropped to 16.7% with Hem07 WT1-CTLs and 6.3% with Hem14 WT1-CTLs (Fig. 4A). We did not see a notable benefit from pretreatment with VEN on THP-1 cells.

Next, we tested pretreatment with VEN on WT1-CTLs with IL-15 maintained in the CTL media during VEN exposure to minimize confounding effects of VEN cytotoxicity on CTLs. CTLs were then washed to remove VEN and IL-15, and AML cells were placed in co-culture with either normal WT1-CTLs or VEN-pretreated WT1-CTLs. Using doses of VEN that increased DNT cytotoxicity in a prior report (100 nM and 200 nM), we observed minimally increased cytotoxicity with VEN-pretreatment of the WT1-CTLs from multiple donors on all 3 AML cell lines (Fig. 4B, Supplementary Fig. 3B) [29]. For OCI-AML2 cells treated with Hem14 WT1-CTLs (E:T 0.5), AML cell viability was 28.3%. If WT1-CTLs were pretreated initially for 24 h with VEN 100 nM or 200 nM and then placed in co-culture (after washing VEN off CTLs), OCI-AML2 viability dropped to 21.3% and 16.5%, respectively (Fig. 4B). Though these values met statistical significance, the magnitude of difference was low. Similar findings were seen when Hem07 and Hem15 WT1-CTLs were co-cultured with OCI-AML2 and OCI-AML3 cells (Fig. 4B, Supplementary Fig. 3B). Notably, we found minimal to no increase in THP-1 cell death when WT1-CTLs were pretreated with VEN. These data suggest that VEN-pretreatment of CTLs only minimally improves CTL cytotoxicity against AML cells and that the effect of VEN is largely on AML cells instead of on CTLs.

### CTL treatment activates the intrinsic/mitochondrial apoptotic pathway in AML cells

**Fig. 5:**
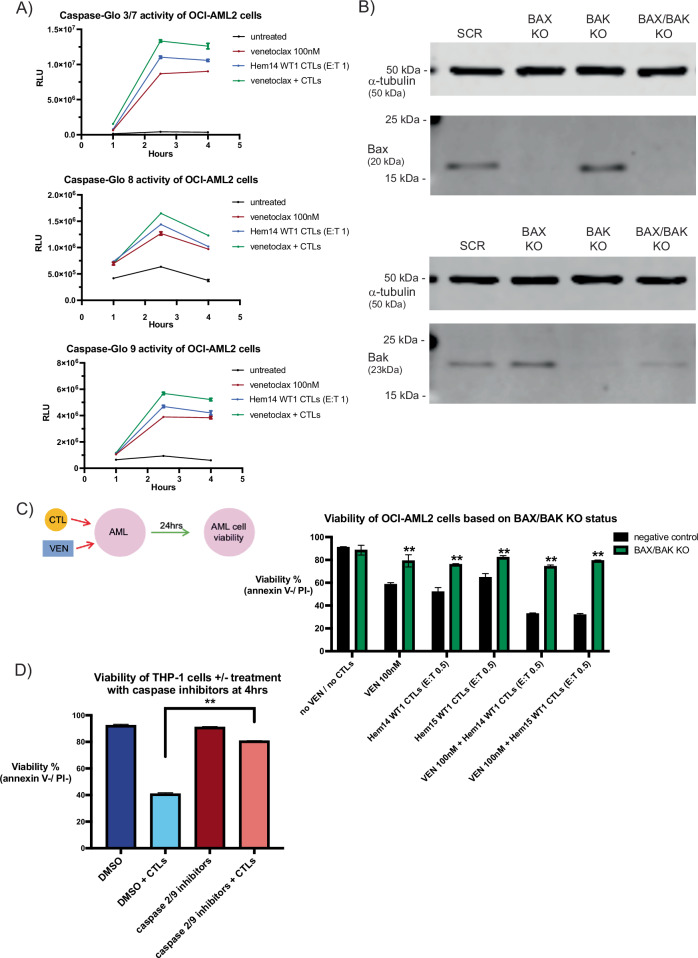
Role of the intrinsic apoptotic pathway in VEN + CTL treatment. **A** OCI-AML2 cells were placed in co-culture with WT1-CTLs (Hem14 donor, E:T 1) ± VEN (100 nM). Prior to co-culture, CTLs were labeled with cell trace violet (CTV). At the indicated time points, CTV-negative (AML cells) were separated from CTV+ cells by FACS (5 min sorting per sample) and CTV-negative cells were immediately placed in the indicated Caspase-Glo buffer for 45 min prior to assessing luciferase activity. Equivalent numbers of CTV-negative cells were sorted for each condition at each timepoint. RLU = relative light unit. **B** Genes for *BAX* and/or *BAK1* were knocked out from OCI-AML2 cells using CRISPR gRNA/Cas9 ribonucleoprotein (RNP) nucleofection. Knockout efficacy was evaluated by western blot for Bax (top) and Bak (bottom) protein, with α-tubulin used as a loading control. SCR = negative control KO (irrelevant scramble sgRNA). Molecular weight markers (15, 25, 50 kDa) are shown. Full western blots available in supplementary file. **C** OCI-AML2 cells based on knockout status (negative control vs *BAX/BAK1* double KO) were treated with VEN (100 nM) ± WT1-CTLs (E:T 0.5) concurrently and AML cell viability was assessed at 24 h using annexin V/PI staining. **D** THP-1 cells were placed in co-culture with WT1-CTLs (Hem14 donor, E:T2) for 4 h. During co-culture, cells were treated with or without inhibitors against caspase 2 and caspase 9 (each at 100 μM). Conditions that did not have caspase inhibitors were treated with an equivalent volume of DMSO as was used for the caspase inhibitor conditions. Results are shown as mean ± standard deviation of *n* = 4 (**A**, **D**) or *n* = 3 samples (**C**) and are representative of 3 (**A**) or 2 (**C**, **D**) independent experiments. Asterisks in (**C**) are comparisons of co-culture with control OCI-AML2 cells vs knockout OCI-AML2 cells. **p < 0.01 using unpaired *t*-test. Schematic of the experimental setup is shown for (**C**).

Classically, the apoptotic pathway of cell death has been divided into an intrinsic (mitochondrial-mediated) pathway and an extrinsic (extracellular-initiated) pathway. In the intrinsic pathway, cellular stressors can directly or indirectly lead to the activation of pro-apoptotic proteins such as Bid and Bim [46]. These ultimately activate the effector proteins Bax and Bak, which dimerize to form pores in the outer mitochondrial membrane [47]. This process leads to the activation of multiple proteins including caspase 9, with caspase 2 also implicated in the intrinsic pathway though its role is not as well-defined as that of caspase 9 [48–50]. In contrast, the extrinsic apoptotic pathway is often triggered by either (a) the perforin/granzyme pathway in which granzymes from T and NK cells directly activate caspases such as caspase 8, or (b) through engagement of cell surface death receptors such as Fas and TRAILR1/R2, which lead to activation of caspase 8 and caspase 10 [47]. Ultimately, both pathways lead to activation of the executioner caspases (caspase 3 and caspase 7) [51]. However, the extrinsic pathway has also been shown to lead to activation of Bid through cleavage by caspase 8 and granzyme B [23, 52]. In order to assess which apoptotic pathways are activated by VEN ± CTL treatment of AML cells, we performed caspase activation studies on OCI-AML2 cells treated with VEN ± WT1-CTLs. WT1-CTLs and VEN led to detectable caspase 3/7 activation by 2.5 h (Fig. 5A). WT1-CTLs activated both the intrinsic and extrinsic apoptotic pathway caspases, as demonstrated by increased caspase 9 and caspase 8 activity, respectively (Fig. 5A). VEN, as expected, led to significantly increased caspase 9 activity. However, VEN also led to increased caspase 8 activity. This finding is consistent with prior reports showing that the BCL-2/BCL-XL inhibitor ABT-737 leads to caspase 8 cleavage measured by western blot in AML cell lines as soon as 1 h post-exposure [53]. Although caspase activity plateaued by 4 h (Fig. 5A), AML cell death continued out to 24 h (Supplementary Fig. 4A).

Given that CTLs could lead to activation of the intrinsic pathway initiator caspase 9 in AML cells, we next assessed the contribution of the mitochondrial apoptotic pathway to CTL-mediated killing of AML cells. In order to assess this, we generated OCI-AML2 cells lacking either Bax, Bak, or both Bax/Bak using CRISPR/Cas9 gene editing with a ribonucleoprotein (RNP) nucleofection approach. This process led to the absence of detectable Bax and Bak protein by western blot in single knockout (KO) OCI-AML2 cells (Fig. 5B). In Bax/Bak double KO OCI-AML2 cells, we noted absence of detectable Bax protein; residual Bak protein remained, albeit at a density of 48% compared to the control sample as quantified by densitometry (Fig. 5B). We performed co-culture assays with these cells 2–4 days after their last round of nucleofection. When compared to OCI-AML2 cells with intact Bax and Bak expression, the Bax/Bak double KO was largely resistant to the effects of VEN (100 nM), WT1-CTLs (E:T 0.5), and the combination of VEN + CTLs (Fig. 5C). These data suggest that the intrinsic/mitochondrial apoptotic pathway plays an important role in CTL cytotoxicity at low E:T ratios and that increased cytotoxicity seen with concurrent VEN + CTLs is largely due to increased activity through the intrinsic/mitochondrial apoptotic pathway, as knockout of Bax/Bak largely eliminates apoptosis through the intrinsic/mitochondrial pathway. Notably, resistance against VEN ± CTLs was primarily seen in the double KO population; there was no resistance against VEN and minimal resistance against CTLs in the Bak KO group and there was moderate resistance to VEN and minimal resistance against CTLs in the Bax KO group (Supplementary Fig. 4B). Although the primary pore-forming complexes in the mitochondria consist of Bax/Bak heterodimers, it is known that both Bax and Bak can homodimerize, providing a potential explanation for the reduced phenotype seen in the single KO groups compared to the double KO group [54, 55]. In addition, AML resistance to VEN has been shown in the setting of loss-of-function of Bax but not Bak, suggesting that the two mitochondrial effector proteins may have unique sensitivities in mediating intrinsic apoptotic cell death [56].

In order to evaluate the contribution of the intrinsic apoptotic pathway in a complementary manner to knockout cells, we chose to utilize inhibitors against caspases associated with the intrinsic apoptotic pathway during AML/CTL co-culture. Caspase 9 and, to a lesser extent, caspase 2 are initiator caspases active in the intrinsic/mitochondrial apoptotic pathway [48–50, 57]. Wild-type THP-1 cells were treated with CTLs ± inhibitors against caspases 2 and 9. Because the caspase inhibitors were cytotoxic to AML cells over the course of a 24 h incubation period, we performed co-culture for 4 h. When THP-1 cells were treated with CTLs for 4 h, viability dropped from 92.3% to 40.9% (Fig. 5D). However, when AML/CTL co-culture was performed in the presence of inhibitors to caspases 2 and 9, viability only dropped from 91% to 80.7% (Fig. 5D), demonstrating the importance of caspase 2/9 activity in mediating CTL cytotoxicity.

We also assessed for a role for death receptor/ligand interactions in mediating CTL killing of AML cells. FasL and TRAIL are the two main death receptor ligands expressed on CTLs and each can initiate extrinsic apoptosis by binding to their respective receptor(s) on-target cells. To assess the contribution of these death receptor/ligand interactions, we utilized blocking antibodies against Fas (to prevent FasL on CTLs from binding Fas on AML cells) or TRAIL (to prevent TRAIL on CTLs from binding its receptors on AML cells). We found no impact on CTL killing of OCI-AML3 cells when antibodies against Fas or TRAIL were used during co-culture (Supplementary Fig. 4C). Notably, we also found that neither high concentrations of exogenous soluble FasL (up to 100 ng/ml) nor soluble TRAIL (up to 50 ng/ml) could induce apoptosis of OCI-AML3 cells (while similar concentrations were able to cause cell death on non-AML cell lines), further suggesting against a role for FasL/TRAIL receptor ligation in mediating CTL-induced AML killing (Supplementary Fig. 4D).

Lastly, we aimed to differentiate intrinsic apoptosis from type II extrinsic apoptosis, as both utilize Bax/Bak dimerization and MOMP to execute apoptosis. However, in the latter, death-receptor signaling (a less likely mechanism in our model per Supplementary Fig. 4C, D) triggers caspase 8 activation and subsequent Bid cleavage, a necessary step for type II extrinsic apoptosis [58]. Thus, type II extrinsic apoptosis contrasts with type I extrinsic apoptosis (which is not Bax/Bak-dependent) and with intrinsic apoptosis, which is caspase 8-independent and can be activated by pro-apoptotic proteins aside from only Bid. To assess for Bid reliance, we generated OCI-AML2 cells lacking Bid (Supplementary Fig. 4E). We found that Bid KO OCI-AML2 cells were only slightly resistant to CTL killing (6% difference in viability between wild-type and Bid KO cells after CTL treatment), suggesting against absolute Bid-dependency and thereby against type II extrinsic apoptosis (Supplementary Fig. 4F). Together, these data across 3 different AML cell lines with complementary assays assessing intrinsic/mitochondrial versus extrinsic apoptotic mechanisms suggest that CTL killing of AML cells is highly dependent on the intrinsic/mitochondrial apoptotic pathway.

### WT1-CTLs can kill primary AML patient samples, including the CD34+/CD38− compartment

**Fig. 6:**
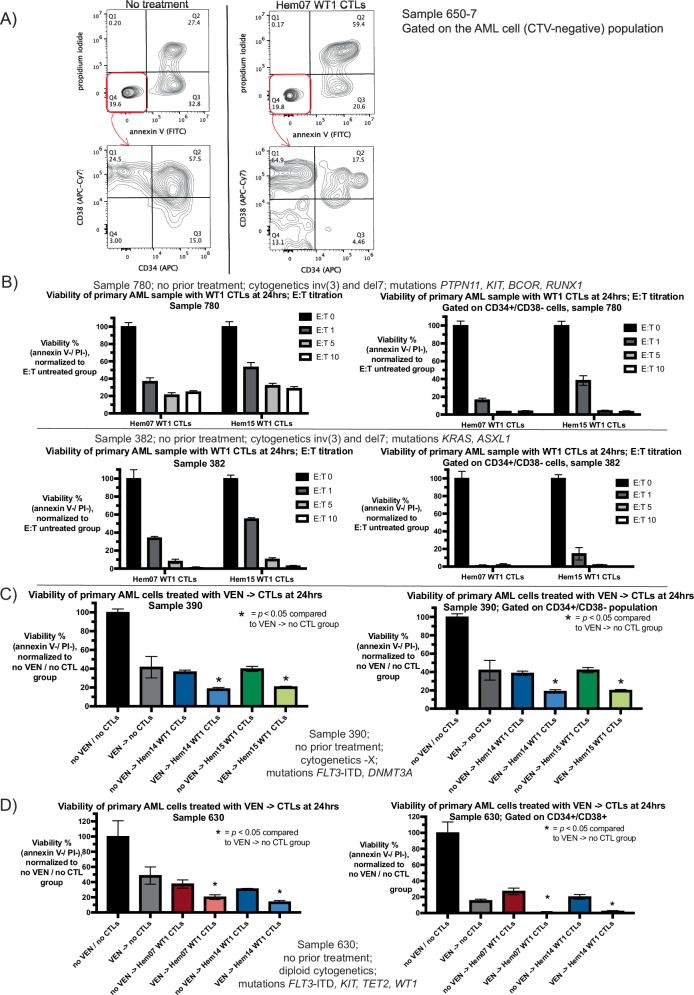
CTLs on primary AML samples with or without VEN-pretreatment. **A** Primary AML cells (sample 650-7) were kept in A221 media for 24 h with or without Hem07 WT1-CTLs (E:T 10). At 24 h, live cell staining was performed with gating as in Supplementary Fig. 1F and the viable AML cells (CTV-negative −> double-negative for annexin V and PI; top row) were evaluated for CD34 (x-axis) and CD38 (y-axis) surface staining (bottom row). Left column shows AML cell viability and CD34/CD38 expression at 24 h in the absence of CTL co-culture; right column shows AML cell viability and CD34/CD38 populations after 24 h of co-culture with CTLs. CD34/CD38 gating was performed using isotype controls. CTV cell trace violet. **B** Primary AML cells (sample 780 and sample 382) were placed in co-culture in A221 media for 24 h with WT1-CTLs from two different donors at various E:T ratios. AML cell viability after 24 h of co-culture is shown on the whole population of AML cells as well as of the CD34+/CD38− population specifically. Viability was compared to the same primary AML cells that were in A221 media for 24 h without CTLs, which was normalized to 100%. **C** and **D** Primary AML cells (**C**: sample 390 and **D**: sample 630) were either pretreated with or without VEN in A221 media for 24 h. After VEN exposure, AML cells were washed three times to remove VEN and then were placed in culture with or without WT1-CTLs for an additional 24 h. At the conclusion of 24 h of co-culture (48 h from experiment initiation), AML cell viability was assessed by annexin V/PI staining and CD34/CD38 surface staining was performed. AML cell viability is shown on the whole population of AML cells as well as of the indicated CD34+ population specifically (**C**: CD34+/CD38− and **D**: CD34+/CD38+). Viability was compared to the same primary AML cells that were in A221 media for 48 h without VEN or CTLs, which was normalized to 100%. Experimental conditions were (**C**) VEN 5 nM and E:T 10:1 and (**D**) and VEN 10 nM and E:T 5:1. Results are shown as mean ± standard deviation of *n* = 3 samples (**B**–**D**). Asterisks are comparisons of VEN treatment alone vs VEN −> CTL treatment; *p < 0.05 using unpaired *t*-test.

In order to assess if the results we found in 3 mutationally diverse AML cell lines can be replicated in primary AML patient samples, we obtained cryopreserved samples from patients with AML who were verified to be HLA-A*02:01 positive by DNA genotyping and had high blast counts (sample characteristics summarized in Table 1). We assessed 14 separate samples obtained from 13 different patients. Age at collection ranged from 26 to 72 years old (median 42), pre-processing sample blast count ranged from 44 to 94% (median 74%), 7/14 samples were of intermediate-risk disease at the time of sample collection (as assessed by European LeukemiaNet 2022 risk stratification) and 7/14 were adverse-risk disease. 4/14 samples came from patients with prior AML-directed chemotherapy including one patient with prior VEN exposure. 5/14 samples were from bone marrow samples, with the remainder coming from peripheral blood. All samples underwent Ficoll gradient processing to obtain mononuclear cells, and an additional 8/14 underwent subsequent CD3/CD19 depletion prior to cryopreservation. After processing, all samples were highly enriched for blast cells (Supplementary Fig. 5, Fig. 6A). In addition, 8/11 samples that had CD34+ blasts on clinical pathology at the time of sample collection had CD34 staining repeated as positive in our assays. WT1-CTLs generated from 3 different donors were able to kill primary AML samples at E:T ratios ranging from 1 to 10, with killing plateauing at an E:T of 5–10 (Fig. 6B shows 2 representative patients). Notably, WT1-CTLs were able to kill CD34+/CD38− AML cells (Fig. 6A, B). Of the 14 samples we assessed, WT1-CTLs from the 3 donors (Hem07, Hem14, and Hem15) led to AML cell death in 12/14 samples, with ≥20% cytotoxicity (≤80% viability post-CTL treatment) used as a cutoff for defining susceptibility to WT1-CTLs (Supplementary Table 1, Supplementary Fig. 6). Of the 6 samples with a detectable CD34+/CD38− population, WT1-CTLs could kill CD34+/CD38− cells in all 6 samples, with preferential killing of CD34+/CD38− AML cells compared to the total population of AML cells in 5/6 samples; sample 390 showed similar viability of CD34+/CD38− cells to that of the total population post-CTL treatment (Supplementary Fig. 6, Supplementary Table 1).

**Table 1:** Characteristics of primary AML samples.

Patient	Age at Collection	Prior treatment	PB vs BM	Morphologic blast% pre-processing	Type of processing (Ficoll vs Ficoll + CD3/CD19 depletion)	CG	Mutational profile	ELN 2022 risk status
432	69	No	PB	94%	CD3-/CD19-	diploid	NPM1, TET2, FLT3-ITD	I
992	26	No	PB	90%	CD3-/CD19-	diploid	NPM1, FLT3-ITD, DDX41, GATA2	I
390	45	No	PB	79%	CD3-/CD19-	-X	FLT3-ITD, DNMT3A	I
114	39	No	PB	71%	Ficoll	diploid	NPM1, FLT3-ITD, DNMT3A	I
988	27	No	BM	83%	CD3-/CD19-	del5	FLT3-ITD, WT1	A
964	44	7 + 3, HiDAC	BM	67%	Ficoll	diploid	FLT3-ITD, IDH2, NPM1	I
118	40	7 + 3, HiDAC	BM	55%	CD3-/CD19-	diploid	NPM1, SRSF2	A
444	72	No	BM	50%	CD3-/CD19-	del5	DNMT3A, IDH2	A
650-7	53	7 + 3 + GO	PB	44%	CD3-/CD19-	inv(3), del7	PTPN11	A
650-1.7	55	7 + 3 + GO, HiDAC + idasanutlin, AZA/ipilimumab/nivolumab, AZA/VEN/GO, FLAG-Ida/VEN/gilteritinib, CYC065/VEN, BIDFA	BM	90%	Ficoll	inv(3), del7	PTPN11	A
382	46	No	PB	79%	CD3-/CD19-	inv(3), del7	KRAS, ASXL1	A
780	36	No	PB	70%	Ficoll	inv(3), del7	PTPN11, KIT, BCOR, RUNX1	A
630	30	No	PB	63%	Ficoll	diploid	FLT3-ITD, KIT, TET2, WT1	I
1988	27	No	PB	77%	Ficoll	*t*(4;9)	FLT3-ITD, NRAS, ABL1	I

*PB* peripheral blood, *BM* bone marrow, *CG* cytogenetics.

*ELN* European LeukemiaNet, *I* intermediate-risk, *A* adverse/poor-risk.

*7* *+* *3 =* infusional cytarabine × 7d + anthracycline × 3d, *HiDAC* high-dose cytarabine.

*GO* gemtuzumab ozogamicin, *AZA* azacytidine.

*BIDFA* twice a day fludarabine + cytarabine, *FLAG-Ida* fludarabine, cytarabine, GCSF, idarubicin.

*CYC065* investigational CDK inhibitor, *VEN* venetoclax.

### Venetoclax-pretreatment followed by WT1-CTLs leads to increased AML cell death using primary patient samples

We subsequently assessed if VEN-pretreatment of AML primary samples leads to increased cell death when followed by CTL co-culture. We chose this pretreatment approach to mimic a clinical scenario in which a patient receives VEN 24 h prior to adoptive T cell infusion in order to avoid the potentially deleterious effects of VEN on CTL viability when VEN + CTLs are delivered concurrently. We evaluated 6 primary AML samples with this approach, and in all 6 samples VEN-pretreatment followed by CTL co-culture led to increased AML cell death (2 representative patients shown in Fig. 6C, D; of the 6 patients, 2 had adverse-risk disease and 4 had intermediate-risk disease). Compared to the AML cell lines, these primary AML samples were more sensitive to VEN (5–10 nM VEN necessary to yield approximately 40–60% cytotoxicity on primary samples compared to 100 nM on OCI-AML2 cells) and less sensitive to WT1-CTLs (E:T 5–10 required to yield cytotoxicity seen with E:T 0.5 in AML cell lines). For sample 390 (*FLT3*-ITD and *DNMT3A* mutations, -X cytogenetics), viability was 41% with VEN 5 nM, 37–40% with WT1-CTLs, and 19–21% with VEN followed by CTLs (Fig. 6C). These viability ranges were similar when gating was performed specifically on the CD34+/CD38− population of cells (Fig. 6C). With sample 630 (*FLT3*-ITD, *KIT*, *TET2*, and *WT1* mutations, diploid cytogenetics), viability of the main leukemic population (CD34+/CD38+) of cells was 15% with VEN 10 nM, 20–27% with WT1-CTLs, and 1–2% with VEN followed by CTLs (Fig. 6D).

### MCL-1 inhibition can be combined with WT1-CTLs in order to target AML cells

**Fig. 7:**
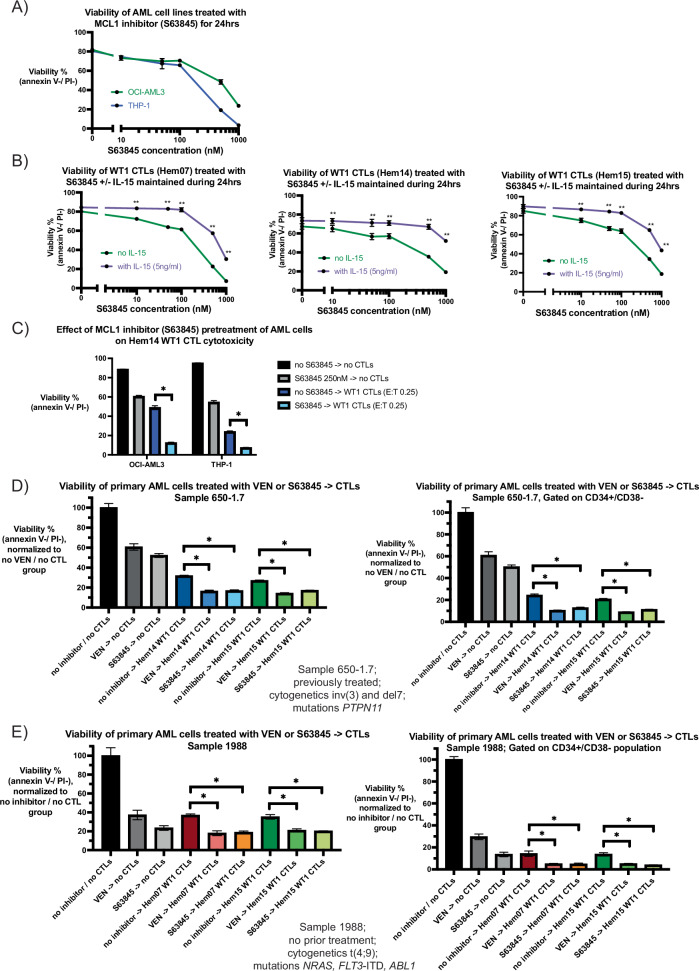
Substitution of an MCL-1 inhibitor for VEN. **A** OCI-AML3 and THP-1 cells were incubated with the MCL-1 inhibitor S63845 for 24 h in A221 media at the indicated concentrations (0, 10 nM, 50 nM, 100 nM, 500 nM, 1000 nM). Viability was assessed at 24 h using annexin V/PI staining. **B** WT1-CTLs from donors Hem07 (left), Hem14 (middle), or Hem15 (right) were first rested for 24 h in CTL media with IL-15 (5 ng/ml). After 24 h of resting, CTLs were transferred to CTL media without IL-15 (green lines) or with IL-15 (5 ng/ml; purple lines) and were treated with varying concentrations of S63845 (0, 10 nM, 50 nM, 100 nM, 500 nM, 1000 nM). Viability was assessed after 24 h of S63845 exposure using annexin V/PI staining. Statistical analyses were performed as a two-way ANOVA followed by post-hoc *t*-test with Bonferroni adjustment for multiple comparisons; ** = adjusted *p* < 0.01. **C** OCI-AML3 and THP-1 cells were either pretreated with or without S63845 250 nM in A221 media for 24 h. After S63845 exposure, AML cells were washed three times to remove S63845 and then were placed in culture with or without WT1-CTLs (Hem14 donor) for an additional 24 h. At the conclusion of 24 h of co-culture (48 h from experiment initiation), AML cell viability was assessed by annexin V/PI staining. Asterisks are comparisons of CTL treatment alone vs S63845 −> CTL treatment; *p < 0.05 using unpaired *t*-test. **D** and **E** Primary AML cells (**D**: 650-1.7 and **E**: 1988) were either pretreated with or without VEN or S63845 in A221 media for 24 h. After inhibitor-exposure, AML cells were washed three times to remove the inhibitor and then were placed in culture with or without WT1-CTLs for an additional 24 h. At the conclusion of 24 h of co-culture (48 h from experiment initiation), AML cell viability was assessed by annexin V/PI staining and CD34/CD38 surface staining was performed. AML cell viability is shown in the whole population of AML cells as well as in the CD34+/CD38− population. Viability was compared to the same primary AML cells that were in A221 media for 48 h without VEN or CTLs, which was normalized to 100%. Experimental conditions in both D and E were VEN 10 nM, S63845 100 nM, and E:T for CTLs 10:1. Statistical analyses were performed for the indicated comparisons per CTL treatment condition as a one-way ANOVA followed by post-hoc testing with an unpaired *t*-test with a Bonferroni adjustment incorporated for significance (*p*/n) where n equals the number of paired comparisons per group (2 comparisons per group), yielding *p < 0.05/2 (*p* < 0.025). Results are shown as mean ± standard deviation of *n* = 3 (**A**, **C**–**E**) or *n* = 4 (**B**) samples and are representative of 2 (**A**–**C**) independent experiments.

Though most patients with AML have VEN-sensitive disease at diagnosis, primary refractory disease can be seen in patients with mutations in *TP53* and/or mutations in activating kinase pathways (such as *RAS, PTPN11, CBL*) [38]. Furthermore, acquired resistance to VEN (defined as an initial response followed by loss of response) is common and associated with the above mutations as well as *FLT3* mutations (ITD more so than TKD) [38]. In some of these mutational subgroups, the anti-apoptotic protein MCL-1 has been shown to play a role in maintaining cell survival [59]. Previous studies have demonstrated that protein expression in AML cell lines for BCL-2 ranks as OCI-AML2 > OCI-AML3 > THP-1 and for MCL-1 ranks as OCI-AML3 > THP-1 > OCI-AML2 [60]. We assessed the sensitivity of THP-1 cells (*RAS*- and *TP53*-mutated) and OCI-AML3 cells (*RAS*-mutated) to the MCL-1 inhibitor (MCL-1i) S63845, as both cell lines were resistant to the BCL-2 inhibitor VEN with no IC50 reached when tested up to doses of 1000 nM of VEN (Fig. 2A). In contrast, both cell lines were sensitive to S63845, with an IC50 of 117.1 nM for THP-1 (95% CI 77.2 nM–170.7 nM) and 263.4 nM for OCI-AML3 (95% CI 161.5 nM–460.9 nM) (Fig. 7A, Supplementary Fig. 7A). Similar to the findings seen with VEN on CTLs, S63845 was cytotoxic to CTLs at doses >100 nM in the absence of IL-15, however IL-15 was not as protective against this toxicity as it was against VEN (Fig. 7B, Supplementary Fig. 7A). We subsequently assessed if S63845-pretreatment of THP-1 and OCI-AML3 led to increased cell death when followed by WT1-CTLs, similar to the approach performed with VEN in Fig. 4A. For OCI-AML3 cells, viability with no CTL nor inhibitor treatment was 88.7%, viability after 24 h of S63845 (250 nM) exposure was 60.5%, and viability after 24 h of co-culture with WT1-CTLs (E:T 0.25) was 48.9% (Fig. 7C). When OCI-AML3 cells were pretreated with S63845 for 24 h (S63845 washed off at 24 h) followed by 24 h of WT1-CTL co-culture, viability dropped to 12.4%. For THP-1 cells, baseline viability was 95.2%, viability after 24 h of S63845 exposure was 54.6%, and viability after 24 h of co-culture with WT1-CTLs was 24% (Fig. 7C). Similar to OCI-AML3 cells, when THP-1 cells were pretreated with S63845 for 24 h followed by 24 h of WT1-CTL co-culture, viability reduced to 7.4%. As was seen with VEN on OCI-AML2 cells, WT1-CTLs activated both caspase 8 and caspase 9 in THP-1 cells, and combined S63845 + CTLs led to higher caspase 3/7 activity compared to either treatment alone (Supplementary Fig. 7B).

Lastly, we assessed if an approach of S63845-pretreatment followed by CTL co-culture led to increased AML cell death utilizing primary AML samples with mutations that have been associated with primary or acquired resistance to VEN. Similar to the approach described in Fig. 6, primary samples were placed intro groups that were untreated for 48 h (no inhibitor/no CTL group), treated for 24 h with VEN or S63845 followed by 24 h of media alone, received 24 h of media alone followed by 24 h of CTL co-culture, or treated for 24 h with an inhibitor (subsequently washed off) followed by 24 h of CTL co-culture. For sample 650-1.7 [*PTPN11* mutation, inv(3) and del7 cytogenetics], viability of the CD34+/CD38− population was 61% with VEN (10 nM) and 50% with S63845 (100 nM), 21–24% with WT1-CTLs (E:T 10:1), 9–10% with VEN followed by CTLs, and 11–13% with S63845 followed by CTLs (Fig. 7D). With sample 1988 [*FLT3*-ITD, *NRAS*, and *ABL1* mutations, t(4;9) cytogenetics], viability of the CD34+/CD38− population was 30% with VEN (10 nM) and 14% with S63845 (100 nM), 13–14% with WT1-CTLs (E:T 10:1), 4–5% with VEN followed by CTLs, and 4–5% with S63845 followed by CTLs (Fig. 7E). Thus, in primary AML samples with mutations predisposing to acquired VEN-resistance after persistent VEN exposure, treatment with an MCL-1i displays similar in vitro activity to VEN in a pretreatment approach with CTLs and can be used either in place of or alternating with VEN to achieve similar levels of AML cell death after CTL exposure.

## Discussion

We demonstrate that a TCR-based approach utilizing WT1-specific CTLs can effectively kill AML cell lines and primary AML cells while sparing normal PBMCs. Based on prior clinical trials in AML, a limitation of this approach has been reduced efficacy in the setting of high leukemic burden [12, 13]. We postulated that the use of BH3 mimetics to lower the cellular apoptotic threshold would allow for more effective T cell-based strategies. In this study, we demonstrate that a combination approach of BH3 mimetic (VEN or S63845) pretreatment followed by exposure to WT1-specific CTLs leads to significantly augmented cell death of leukemic cells in an additive manner while sparing toxicity of the BH3 mimetics on CTLs due to the temporal separation of VEN and CTL exposure. Using Bax/Bak knockout AML cells as well as caspase inhibitors and blocking antibodies, we demonstrate that, mechanistically, the extrinsic pathway is not a significant contributor to this combinatorial efficacy. Rather, our data suggests that the combined effect of BH3 mimetics and CTLs is highly dependent on activation of the intrinsic/mitochondrial apoptotic pathway in the AML cells.

We chose to utilize pMHC-specific CD8+ CTLs rather than NK cells, DNTs, or CAR-T cells for our proof-of-concept studies of VEN + cell therapy for multiple reasons. A pMHC-specific CD8+ T cell approach has the most clinically-proven benefit in AML of delivering a cell product that can persist in circulation while also maintaining a central memory phenotype with inducible cytotoxicity [11, 12, 42]. Furthermore, we chose to use WT1-CTLs as WT1 is the most extensively studied pMHC target in AML, demonstrating efficacy against a broad repertoire of primary patient samples in vitro as well as both safety and efficacy in human clinical trials [11, 12]. In contrast, other common tumor-associated CTL targets such as PRAME and NY-ESO-1 are not expressed as commonly as WT1 in AML cell lines and primary samples and often require pretreatment with a hypomethylating agent or histone deacetylase inhibitor to induce sufficient target expression for CTL recognition [61–63]. DNTs and NK cells lack the target specificity of CD8+ CTLs, and DNT killing of AML cells is hypothesized to be mediated through receptors shared on NK cells and subsets of T cells, such as NKG2D and DNAM-1 [29]. CD8+ CTLs often express these NK-associated receptors found on DNTs [64, 65]. Thus, pMHC-specific CD8+ ETC products have the advantage of potentially killing AML cells in a target-specific (pMHC-dependent) and MHC-independent fashion.

The WT1-specific CTLs utilized in this study demonstrated significant cytotoxicity against high-risk AML populations that typically are more resistant to conventional therapy with chemotherapy and/or VEN, including against *TP53*-mutated cells (THP-1), *PTPN11*-mutated or *RAS*-mutated cells (THP-1, OCI-AML3, multiple primary samples), and primary samples with inv(3) cytogenetics [38, 66, 67]. Furthermore, these WT1-CTLs effectively killed AML cells with a CD34+/CD38− immunophenotype, a cell compartment which is classically enriched with leukemia-initiating cells [(LICs) or leukemic stem cells (LSCs)] [68]. LICs/LSCs are considered chemoresistant compared to rapidly dividing blast cells and are believed to mediate disease relapse [69]. Although WT1-CTLs demonstrated cytotoxicity against multiple clinically relevant populations of AML cells, this study was designed to investigate a possible means of addressing the challenge of high leukemic burden that can reduce CTL efficacy – a significant barrier in prior clinical studies of ETC/TCR approaches for AML as well as for non-AML T-cell-based therapeutic approaches (such as for CD19 bispecific and CAR-T treatments) [13, 70, 71]. We chose BH3 mimetics as a means of lowering the apoptotic threshold of target cells given the proven clinical safety, efficacy, and known pharmacology of the BH3 mimetic VEN for patients with AML. VEN is an oral, daily-dosed BCL-2 inhibitor, with plasma levels peaking approximately 8 h post-dose and returning to pre-dose levels by approximately 24 h [72]. Furthermore, VEN has been shown to be rapidly effective even after a single dose, with in vivo data from patients with chronic lymphocytic leukemia (CLL) treated with VEN demonstrating an increase in apoptotic CLL cells as early as 6 h post-dose [73].

Multiple studies have suggested that VEN is well-tolerated by T cells, in contrast to conventional chemotherapy, permitting combination therapy with CD8+ CTLs. In one study, CD8+ T cells were activated with anti-CD3/CD28 antibodies and incubated with VEN as high as 3 mM for 24 h; no significant decrease in T cell viability was seen [30]. Similarly, VEN was not cytotoxic against DNTs over 18 h of exposure up to a concentration of 2μM [29]. However, in a study primarily of VEN and NK cells, toxicity against CD8+ T cells was also studied [32]. It was found that VEN and S63845 were each highly cytotoxic to T cells, with >50% cytotoxicity seen at 256 nM, while the addition of IL-2 and anti-CD3/CD28 activation beads to T cells mitigated any cytotoxicity in the presence of up to 1024 nM of either inhibitor [32]. Similarly, it has been shown that both VEN and a different MCL-1i (AZD5991) display dose-dependent toxicity on CAR-T cells [33]. In our studies, we found that ETC-generated CTLs were susceptible to VEN and S63845, primarily at doses >100 nM, and the use of IL-15 during VEN exposure diminished toxicity. Although exogenous means of T cell activation (such as exposure to anti-CD3/CD28 antibodies/beads or IL-2/IL-15) can protect against VEN-mediated toxicity, the applicability of these strategies during T cell infusion clinically is questionable. As we demonstrated, T cells that are rested in IL-15 for 24 h and then placed in VEN without IL-15 for 24 h lose the protective effect of IL-15, and thus IL-15 appears to be required throughout the period of VEN exposure. A clinical scenario combining VEN concurrently with infused CD8+ T cells may therefore require constant IL-2 or IL-15 exposure, which may not be clinically feasible and will likely bring significant additional cytokine-induced toxicities [5, 74, 75]. Similar to our findings, a study of CD19 CAR-T cells with both VEN and S63845 in vitro demonstrated that concurrent treatment of lymphoma cells with BH3 mimetic + CAR-T leads to significant toxicity of the BH3 mimetic on CAR-T cells, and pretreatment with the BH3 mimetic followed by CAR-T cells avoided this toxicity [76]. In vivo studies of the effects of VEN on T cell viability vary considerably, though emerging data continues to support that, at least in the CAR-T field, BCL-2 inhibition is toxic to T cells during concurrent administration and requires either temporal separation or engineering CAR-T cells to overexpress BCL-2 or BCL-XL [33, 77–79]. Overall, these data are compatible with our findings in which VEN was significantly cytotoxic against resting CTLs in isolation (Fig. 2B). However, during co-culture with AML cells, the WT1-CTLs were relatively protected against the cytotoxic effects of VEN (Supplementary Fig. 2D), suggesting that during active killing and TCR/CD3 stimulation, higher doses of VEN are tolerated by CTLs. This relative protection by CTLs against VEN during active CTL killing may be due to multiple reasons, including local secretion of IL-2 by activated CTLs leading to autocrine/paracrine signaling; IL-2 (like IL-15) has been shown to lead to BCL-2 upregulation, which can lead to better tolerance of the BCL-2 inhibitor VEN [80].

For these reasons, we chose to temporarily separate VEN from CTLs to avoid toxicity and model a clinical scenario in which VEN is given to a patient the night before CTL infusion. Mechanistically, this approach supports the hypothesis that BH3 mimetics like VEN and S63845 prime AML cells to apoptosis when a secondary insult arrives, and thus BH3 mimetics may be more efficacious when present before or during, rather than following, treatment with a secondary agent. Furthermore, the BH3 mimetic can also be intermittently re-dosed (as opposed to continual daily dosing as is performed for most currently approved VEN dosing regimens) to periodically re-activate the intrinsic apoptotic pathway in AML cells engaging with infused CTLs, or the BH3 mimetic can be “pre-dosed” for greater than one day prior to CTL infusion to potentially achieve a greater depth of mitochondrial apoptotic priming. For the VEN-sensitive AML cell line OCI-AML2, pretreatment with VEN followed by CTLs (E:T 0.5) led to AML cell death similar in magnitude to that seen with CTLs alone at higher E:T ratios (E:T 5), suggesting that pretreatment of AML cells with VEN shifted the E:T ratio equation in favor of the effector T cells. OCI-AML3 and THP-1 cells were less sensitive to VEN, likely due to preferential reliance on MCL-1 rather than BCL-2 for survival. However, substitution of VEN with S63845 in a pretreatment strategy produced similar results in OCI-AML3 and THP-1 as was seen with VEN on OCI-AML2 cells, suggesting that the approach of BH3 mimetic followed by CTL co-culture can be conserved across multiple AML cell phenotypes based on the BH3 mimetic that they are susceptible to. Furthermore, this was reproduced with additive efficacy in primary patient AML samples from multiple different patients with a variety of mutational changes. These data suggest that this pretreatment approach can be applied to most AML patients, with the choice of BH3 mimetic largely driven by a patient’s AML mutational profile. A pretreatment approach in which BH3 mimetic use is temporally restricted to pre-CTL infusion may be of particular interest for the development of MCL-1 inhibitors, for which clinical use has been significantly hindered by the occurrence of toxicities seen with more frequent dosing [81, 82]. Lastly, although the focus of our study was AML, we suspect that a similar approach with different BH3 mimetics (such as targeting BCL-XL) preceding CTL infusion may demonstrate efficacy in solid tumor models, as the concept of apoptotic priming is conserved among tumor types and solid tumors have been shown to be sensitive to MCL-1 and/or BCL-XL inhibitors [83].

The mechanism of cooperative activity between VEN and CTLs appeared to be mediated primarily through simultaneous activity of the intrinsic/mitochondrial apoptotic pathway. In both OCI-AML2 and THP-1 cells, WT1-CTLs activated an intrinsic apoptotic pathway initiator caspase (caspase 9). To follow-up these findings, we knocked out Bax/Bak, the two pro-apoptotic effector BCL-2 family of proteins that are necessary for mitochondrial apoptosis by causing MOMP upon dimerization. We found that both CTL and VEN efficacy were significantly reduced in Bax/Bak double KO AML cells and that the combination of VEN + CTLs did not overcome this deficit, indicating the importance of mitochondrial apoptosis to CTL cytotoxicity of AML cells. However, it is known that both intrinsic apoptosis and type II extrinsic apoptosis can both utilize MOMP for initiating apoptosis and thus the caspase 9 activation and Bax/Bak results could not differentiate intrinsic apoptosis from type II extrinsic apoptosis [58, 84]. Although both pathways can lead to Bid cleavage and subsequent Bax/Bak dimerization and MOMP, they differ in their initiating caspases [85]. To differentiate intrinsic apoptotic pathway dependency from type II extrinsic apoptosis, we performed co-culture assays in the presence of inhibitors against caspase 2/9 (the intrinsic pathway initiator caspases which are not necessary for type II extrinsic apoptosis). We found that inhibition of caspase 2/9 nearly completely eliminated CTL cytotoxicity of AML cells. Because type II extrinsic apoptosis is hypothesized to occur through death receptor engagement leading to caspase 8 activation and subsequent caspase 8-mediated Bid cleavage, we assessed the role of FasL/Fas and TRAIL/TRAIL-R interaction and found no impact on CTL killing. It has previously been shown that TNFα, another activator of extrinsic apoptosis, does not lead to AML cell death [86]. Interestingly, high concentrations of exogenous soluble FasL or soluble TRAIL could not induce apoptosis in AML cells either, further reducing the likelihood that CTL membrane-bound FasL or TRAIL could induce apoptosis in AML cells. Lastly, we found that CTL killing of AML cells was not Bid-dependent, suggesting against the Bid-dependent type II extrinsic apoptotic pathway. Thus, our findings of a Bax/Bak-dependent, caspase 2/9-dependent, FasL/TRAIL-independent, Bid-independent process suggest the importance of intrinsic/mitochondrial apoptosis in mediating CTL killing of AML cells at the E:T ratios utilized in our studies. We hypothesize that TCR activation leads to perforin/granzyme release by CTLs, subsequent pro-apoptotic protein cleavage and/or direct caspase 9 activation within the AML cells by granzyme B, followed by Bax/Bak-mediated MOMP and apoptosis. Notably, the precise mechanisms by which CTL-derived granzyme B release can mediate target cell mitochondrial apoptosis and the relative necessity of Bid/Bax/Bak in this process have been extensively studied but remain incompletely understood [87, 88]. We suspect that the caspase 8 activation seen in our caspase activation studies (Fig. 5A) is a non-essential process for CTL killing of AML cells and is not mediated by death receptor engagement but rather by (a) downstream caspases that have been shown to cleave caspase 8, such as caspase 6 and/or (b) by granzyme B itself, which has also been shown to cleave caspase 8 [89–91].

The extrinsic apoptotic pathway (which does not require Bak/Bak or caspase 2/9) could not compensate for inhibition of the intrinsic apoptotic pathway in our findings, contrasting with the classical model in which CTLs primarily kill tumor cells through activation of extrinsic apoptosis. This is consistent with prior findings of NK cells on various tumor cell lines, which have shown that at low E:T ratios NK cell killing is mediated through the intrinsic apoptotic pathway whereas at higher E:T ratios the intrinsic apoptotic pathway is not as necessary [32]. Given the hypothesis that tumor-reactive CTLs often experience a low E:T ratio in vivo due to higher local tumor burden, simultaneous activation of the intrinsic apoptotic pathway by BH3 mimetics and CTLs may have significant in vivo relevance. In the intrinsic/mitochondrial apoptotic pathway, the concept of an “apoptotic cliff” exists, in which a cancer cell can be pushed or primed towards undergoing MOMP [25, 92]. BH3 mimetics are a prototypical class of drugs that can prime a cell towards falling off this “cliff” when a subsequent intrinsic apoptotic insult occurs [83]. Although these subsequent insults are often thought of as chemotherapeutic agents, radiation, or small molecule inhibitors, as previously stated it has been shown that NK cells can also prime cells towards intrinsic apoptosis [32]. Our data suggests that BH3 mimetics can push AML cells closer to their apoptotic cliff, and because CTLs can activate intrinsic/mitochondrial apoptosis in AML cells, CTLs can also be a potent subsequent mitochondrial insult that can commit a cell to MOMP and cell death.

In conclusion, our study strongly supports the use of BH3 mimetics as T cell partners due to their potential for augmenting T cell-mediated apoptosis. This model was evaluated across an array of AML cells originating from cell lines and primary AML samples from various mutational, cytogenetic, and treatment-status backgrounds. Based on findings of combinatorial activation of the intrinsic/mitochondrial apoptotic pathway in AML cells, we propose a rational clinical trial design approach for adoptive cell therapy in AML in which a patient’s AML cells are ‘conditioned’ for increased sensitivity to subsequent CTL-based adoptive cell therapy by treatment with BH3 mimetics *prior* to infusion of AML-specific CTLs, thus avoiding potential toxicity of the BH3 mimetic on CTLs and delivering equivalent killing as concurrently administered strategies.

## Materials and methods

### Cell lines and primary AML samples

OCI-AML2 and OCI-AML3 cells were obtained from Deutsche Sammlung von Mikroorganismen und Zellkulturen (Braunschweig, Germany) and THP-1 cells were obtained from the American Type Culture Collection (Manassas, Virginia). Cell lines were authenticated by short tandem repeat analysis, performed at the University of Texas at MD Anderson Cancer Center (Houston, TX) Cytogenetics and Cell Authentication Core. HLA-A genotype of the AML cell lines was obtained from the TCLP online database [93]. Notable mutational and cytogenetic abnormalities of the AML cell lines were obtained from the Cellosaurus and DepMap online databases [94, 95]. AML cell lines were cultured in RPMI 1640 (catalog 01-0100DK, Thermo Fisher Scientific, Waltham, MA) supplemented with 10% fetal bovine serum (vol/vol; Sigma–Aldrich), 4 mM L-glutamine (Corning, NY, catalog 25-005-CV), 1 mM pyruvate (Thermo, catalog 11360-070), 1% penicillin/streptomycin (vol/vol; Thermo, catalog 15140-122, stock 10,000 U/ml penicillin and 10,000 µg/ml streptomycin), and 1× non-essential amino acids (Corning, catalog 25-025-CI). This media formulation was named A221 media. All cell lines were verified to be negative for mycoplasma using the LookOut Mycoplasma PCR-based detection test (Sigma–Aldrich). Primary samples were collected from patients with AML who were treated at The University of Texas MD Anderson Cancer Center and who had consented to donate their peripheral blood and/or bone marrow for research protocols approved by the MD Anderson Institutional Review Board and originated from patients who were HLA-A*02:01+ as verified by DNA genotyping. Cryopreserved primary AML samples were thawed for experimental use as previously described and kept in A221 media without any additional cytokines [96].

### Generation of CTLs

WT1-specific CTLs were generated from PBMCs obtained from healthy donors from HemaCare (Northridge, CA) per the endogenous T cell (ETC) protocol utilizing peptide-pulsed DCs and IL-21 priming as previously described [43, 97]. All PBMC donors were HLA-A*02:01-positive. The HLA-A*02:01-restricted WT1 peptide RMFPNAPYL (amino acids 126–134) was obtained from Elim Biopharmaceuticals (Hayward, CA). Phycoerythrin (PE)-conjugated tetramer to detect TCRs binding the described WT1 pMHC complex was custom prepared by the Immune Monitoring Lab at the Fred Hutchinson Cancer Research Center (Seattle, WA). Control tetramer staining was performed with a PE-conjugated tetramer against an HLA-A*02:01-restricted NY-ESO-1 peptide (SLLMWITQC) obtained from MBL Life Science.

CD8+ WT1-tetramer-specific CTLs were sorted on a BD FACSAria Fusion Cell Sorter or a BD FACSAria III Cell Sorter (BD Biosciences, San Jose, CA) at the University of Texas at MD Anderson Flow Cytometry and Cellular Imaging Core. These CTLs were then expanded per a rapid expansion protocol (REP) as previously described [43]. After the first REP cycle (10–13 days), CTLs were re-sorted as above to obtain a population of CD8+ cells that was >95% tetramer-specific for the WT1 pMHC target and then re-expanded per the REP protocol for an additional 10–13 days (REP2). REP cycles for growing CTLs consisted of anti-CD3, IL-2, and irradiated feeder cells as previously described without any other additional cytokines or compounds added [43]. CTLs were generally grown to quantities of 50–100 million cells and then cryopreserved for further use. The primary type of media used during CTL generation is termed CTL media and is the same as A221 media with the following modifications: no additional pyruvate, no additional non-essential amino acids, plus the addition of 100 ul of beta-mercaptoethanol per 1 L of media. CTL media was used at all steps during CTL generation aside from the dendritic cell generation phase, which uses AIM-V-based media [43].

### Co-culture cytotoxicity assays

For co-culture assays in which WT1-CTLs were mixed with target cells (normal donor PBMCs, AML cell lines, or primary AML samples), WT1-CTLs were thawed from cryopreservation (liquid nitrogen, approximately −140 to −160 degrees Celsius) by rapidly shaking them in a water bath (37 degrees Celsius). When the cells were thawed enough to be inverted in a cryovial, they were immediately transferred to 10 ml (per 1 ml of frozen cells) of warm (37 degrees Celsius) RPMI media. CTLs were then centrifuged at 400 × *g* for 5 min and the supernatant was aspirated. CTLs were resuspended in CTL media containing 5 ng/ml of IL-15 (R&D Systems, Minneapolis, MN) at a concentration of 1–2 million CTLs/ml, aliquoted in a 48-well plate (Thermo Fisher Scientific, Waltham, MA), and rested overnight in an incubator set to 37 degrees Celsius. On the following day, CTLs and target cells were quantified prior to co-culture on a hemocytometer using trypan blue exclusion as marker of live cells. In order to perform co-culture assays, either effector cells (CTLs) or target cells were tagged with cell trace violet (CTV; Thermo) in order to separate the populations during flow cytometry data analysis. For most experiments, target cells were chosen as the population to be stained. If target cells were the population being stained in a specific experiment, they were centrifuged at 300 × *g* for 3 min and the supernatant was aspirated. Cells were washed once with PBS. Cells were then resuspended in PBS containing 1500 nM of CTV and kept in the dark for 15–20 min. After 15–20 min, FBS was directly added to the cells at a 1:1 ratio of FBS to PBS in order to stop the CTV staining process. Cells were then centrifuged immediately at 300 × *g* for 3 min and then washed twice in A221 media. Target cells and CTLs were then mixed at the indicated effector:target (E:T) ratios in A221 media and placed in a 96-well round bottom plate (Thermo). If CTLs were the population being stained with CTV, the same above staining process was performed with the modification of centrifugation speed being 400 × *g* rather than 300 × *g*.

After the indicated duration of co-culture, the 96-well plate was centrifuged at 500 × *g* for 3 min. After removing the supernatant, cells were washed once with PBS and centrifuged again. After this wash, cells were resuspended in annexin binding buffer (Biolegend, San Diego, CA, catalog 422201) containing annexin V-FITC (Biolegend, catalog 640945) and propidium iodide (PI; Thermo, catalog P3566). For experiments in which the target cells were primary AML samples, after the PBS wash, cells were stained with anti-CD34 and anti-CD38 antibodies kept in PBS containing 2% FBS for 15 min in the dark. After 15 min, the cells were centrifuged and after discarding the supernatant they were resuspended in the annexin/PI mixture described above. Cells were analyzed on a NovoCyte flow cytometer (Agilent Technologies, Hayward, CA) or a BD LSRFortessa flow cytometer (BD Biosciences). Viability of the target cell population was analyzed using FlowJo 10 software. Gating strategy is shown in Supplementary Fig. 1F. For experiments in which the target cells were AML cell lines or PBMCs, viability was calculated as the percentage of cells negative for both annexin V and PI. For experiments in which the target cells were primary AML cells, experiments were performed on the NovoCyte flow cytometer utilizing the absolute count setting function and viability was normalized to the primary AML samples that received no CTLs nor inhibitors; normalization was performed because baseline viability of the primary AML samples after thawing was generally <50%.

For co-culture between WT1-CTLs and T2 cells, T2 cells were pulsed with varying concentrations of WT1 (amino acids 126–134; RMFPNAPYL) for 30 min at 37 degrees Celsius. T2 cells are HLA-A*02:01 positive and TAP-deficient (transporter associated with antigen processing). After peptide-pulsation, T2 cells were washed twice and then placed in culture with WT1-CTLs for 24 h. Viability was assessed at 24 h by annexin V and PI staining as performed for AML cells.

For co-culture experiments with blocking antibodies, the following conditions were applied: for Fas/FasL interaction blockade, an antibody against Fas was used (clone A16086F, Biolegend). This antibody clone has demonstrated blocking ability at concentrations ≥5 μg/ml [98]. AML cells were pre-incubated with the anti-Fas antibody at a concentration of 12.5 μg/ml for 30 min at 37 degrees Celsius, following which the AML cells with the anti-Fas-containing media were placed into co-culture for 4 h with WT1-CTLs, for a final concentration of anti-Fas of 6.25 μg/ml. For TRAIL interaction blockade, an antibody against TRAIL was used (clone RIK-2, Biolegend) and WT1-CTLs cells were pre-incubated with the antibody at a concentration of 40 μg/ml for 30 min at 37 degrees Celsius, following which the CTLs with the anti-TRAIL-containing media were placed into co-culture for 4 h with AML cells, for a final concentration of anti-TRAIL of 20 μg/ml. The RIK-2 clone has demonstrated blocking ability at concentrations ≥10 μg/ml [99, 100]. Isotype controls for anti-Fas and anti-TRAIL were used as controls for nonspecific antibody-mediated receptor blockade at the same concentrations as indicated above. AML cell viability was assessed at 4 h by annexin V and PI staining as described above.

### Chemicals and Caspase-Glo assays

Venetoclax (VEN, ABT-199) and S63845 were purchased from Selleck Chemicals (Houston, TX). The IC50 in Figs. 2 and 7 was calculated using GraphPad Prism using a nonlinear regression with a variable slope. Caspase-Glo assays for caspase 3/7, caspase 8, and caspase 9 were all purchased from Promega (Madison, WI). For caspase assays, AML cell lines were treated with a BH3 mimetic and/or CTLs. CTLs were stained with CTV prior to incubation with AML cells, as described above for co-culture assays. At the indicated time points, CTLs were removed from the co-culture with AML cells through fluorescence-activated cell sorting (FACS; less than 5 min per sample per sort) and equal numbers of AML cells were added into replicate wells into white 96-well plates (Thermo). In addition, AML cells that were untreated or only treated with a BH3 mimetic still were processed through the same cell sorter as the co-culture samples so that all groups were similarly exposed to the physical stresses of FACS prior to assessing caspase activity. Caspase-Glo reagent containing luciferase and the specific caspase substrate were added to the AML cells per the manufacturer’s instructions (Promega). After 45 min of incubation on a shaker, plates were read on a VICTOR Nivo multimode plate reader (PerkinElmer, Waltham, MA). Inhibitors of caspase 2 (Z-VDVAD-FMK) and caspase 9 (Z-LEHD-FMK) were purchased from R&D Systems, reconstituted in DMSO, and further diluted to a final concentration of 100 μM in media per the manufacturer’s instructions. For co-culture experiments with caspase inhibitors, conditions that did not have caspase inhibitors were treated with an equivalent amount of DMSO as was used in the caspase inhibitor conditions to control for cellular toxicity from DMSO itself. For death receptor experiments, soluble FasL and soluble TRAIL were obtained from Thermo Fisher.

### Generation of knockout cell lines

Gene knockout kits against *BAX*, *BAK*, and *BID* as well as Cas9 from *Streptococcus pyogenes* (SpCas9) were purchased from Synthego (Redwood City, CA). sgRNA sequences are detailed below. Lyophilized sgRNA was resuspended in TE (Tris-EDTA) buffer per the manufacturer’s instructions. OCI-AML2 cells were transfected per a ribonucleoprotein (RNP) strategy of SpCas9 combined with sgRNA against the gene of interest. For negative control cells, SpCas9 was paired with a non-targeting negative control sgRNA obtained from Synthego. RNP complexes were incubated for 15 min at 37 degrees Celsius prior to addition to OCI-AML2 cells. OCI-AML2 cells were washed once in PBS and then resuspended in nucleofection SF buffer obtained from Lonza (Basel, Switzerland) containing the RNP complex. The cell suspension was then immediately placed in a Lonza Amaxa 4D-Nucleofector X Unit using a 96-well plate format, and nucleofection was performed using the FF-120 protocol. OCI-AML2 cells were placed back into A221 media and either underwent repeated rounds of nucleofection or were assessed for BAX/BAK or BID protein expression by western blot. In order to create near-complete knockouts in a bulk population of cells, we performed several rounds of RNP nucleofection because these mitochondrial proteins are intracellular, precluding cell sorting for live knockout (KO) cells. We noted that protein expression by western blot would return by 4–5 days post-RNP nucleofection, suggesting that wild-type cells remained, which could outgrow the knockout population, and thus experiments were performed within 4 days of the terminal nucleofection. The control sample underwent the equivalent number of Cas9/nucleofection treatments with a control scramble gRNA as detailed above.

**Table:** 

Gene	sgRNA
*BAX*	1) U*U*C*UGACGGCAACUUCAACU
	2) C*A*C*CUUGAGCACCAGUUUGC
	3) C*U*G*CAGGAUGAUUGCCGCCG
*BAK*	1) A*C*U*UCACCAAGAUUGCCACC
	2) C*U*C*CUACAGCACCAUGGGGC
*BID*	1) C*U*U*UCCCCUCAGGUCAACAA
	2) C*C*G*CAGAGAGCUGGACGCAC
	3) C*U*C*AUCGUAGCCCUCCCACU

### Western blot

Equal numbers of cells were lysed in 1× Laemmli buffer (Bio-Rad, Hercules, CA). Whole cell proteins were resolved by SDS-PAGE then transferred onto a PVDF membrane (Bio-Rad; 0.2μm). The membrane was blocked with 5% non-fat dry milk in 0.1% Tween-20/PBS for 1 h at room temperature and then incubated with the primary antibodies detailed below in 1% non-fat dry milk in 0.1% Tween-20/PBS at 4 degrees Celsius overnight. After washing with 0.1% Tween-20/PBS three times, the membrane was incubated in the secondary antibodies detailed below in 1% non-fat dry milk in 0.1% Tween-20/PBS for 1 h at room temperature, followed by washing three times. The membrane was scanned using an Odyssey CLx Imager (LI-COR Biosciences, Lincoln, NE) and normalized to the density of α-tubulin in the corresponding samples. Densitometric analysis was performed using ImageJ software [101]. Chameleon Duo Pre-stained Protein Ladder (LI-COR) was used as the ladder. Raw western blot images are shown in supplemental data.

**Table:** 

Antibody, dilution	Company	Catalog
Bax, 1:1000	Cell Signaling Technology, Danvers, MA	2772; rabbit
Bak, 1:1000	Cell Signaling Technology	12105; rabbit
Bid, 1:1000	Cell Signaling Technology	2002; rabbit
Alpha-tubulin, 1:10,000 (for BAX/BAK blots) 1:5000 (for BID blots)	Abcam, Cambridge, UK	ab7291; mouse
Goat anti-rabbit IgG, 1:5000	Invitrogen/Thermo	SA5-10036
Goat anti-mouse IgG, 1:5000	Invitrogen/Thermo	35518

### Flow cytometry

As described above, flow cytometry was performed on a NovoCyte flow cytometer (Agilent Technologies, Hayward, CA) or a BD LSRFortessa flow cytometer (BD Biosciences). Raw data files were analyzed using FlowJo 10 software. Compensation was performed using single-color controls. Antibodies used in this study are listed below.

**Table:** 

Antibody/fluorophore	Company	Catalog
CD3, Brilliant Violet 785 (BV785)	Biolegend	344842
CD4, BV570	Biolegend	300534
CD8, APC	Biolegend	344722
CD16, PE	Biolegend	302008
CD34, APC	Biolegend	343509
CD38, APC-Cy7	Biolegend	356615
CD45, BV785	Biolegend	304047
CCR7, APC	Biolegend	353214
CD45RA, PE-Cy7	Biolegend	304125
HLA-A2, PE	Biolegend	343305
MHC class I, APC	Biolegend	311409
TCRα/β, Alexa Fluor 488	Biolegend	306711
Isotype control, APC, mouse IgG1κ	Biolegend	400121
Isotype control, APC, mouse IgG2aκ	Biolegend	400219
Isotype control, APC-Cy7, mouse IgG1κ	Biolegend	400127
Isotype control, BV785, mouse IgG1κ	Biolegend	400169
Isotype control, PE, mouse IgG2bκ	Biolegend	401207
Isotype control, Alexa Fluor 488, mouse IgG1κ	Biolegend	400132

### Statistical analysis

Statistical analyses were performed using Microsoft Excel software or GraphPad Prism version 9.2.0. Data is shown as mean ± standard deviation. Comparisons between groups were performed as specified in the figure legends.

## Supplementary information

Supplementary Figures 1–7 and Supplementary Table 1

Supplementary File With Full Western Blots

## Data Availability

All data are available in the main text or the supplementary material.
