# Supplementary material for: BH3 mimetics augment cytotoxic T cell killing of acute myeloid leukemia via mitochondrial apoptotic mechanism
Source: Cell Death Discov. 2025 Mar 26;11:120. doi: 10.1038/s41420-025-02375-2 (PMC11947210; doi:10.1038/s41420-025-02375-2)
Supplement: Supplementary file 1 — Supplementary Figures 1–7 and Supplementary Table 1 [file 41420_2025_2375_MOESM1_ESM.docx]

**List of supplementary materials**

Fig. S1-S7: Supplementary Figures 1-7

Table S1: Supplementary Table 1

**Supplementary Figure 1**

**
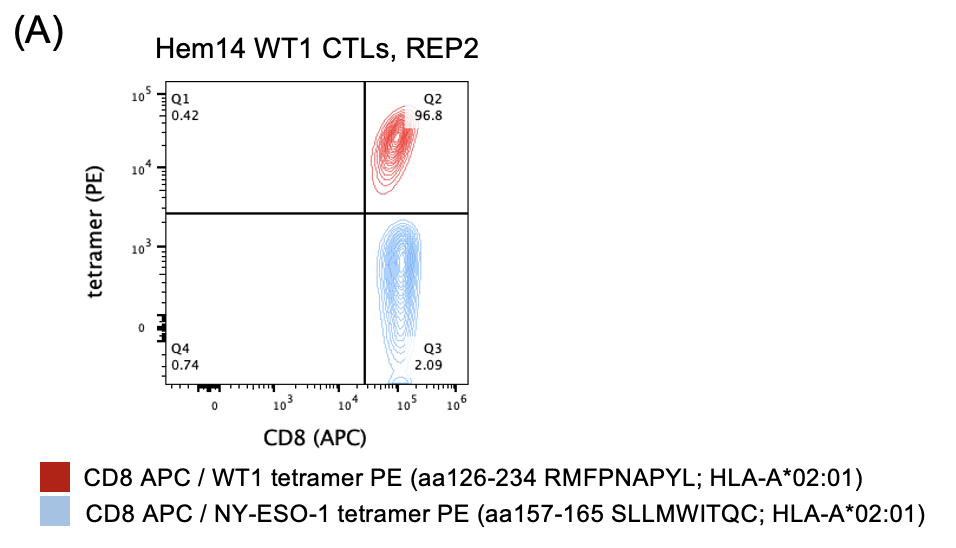
**

(D)

(E)

(F)

**Fig. S1. Additional characterization of WT1-CTLs**

1. Tetramer staining for a control tetramer (NY-ESO-1, blue) and for the WT1 tetramer (red) on ETC-generated CTLs from donor Hem14. Gating strategy for tetramer stain (and subsequent staining of CTLs) involved first gating on FSC-A high, SSC-A low cells to exclude most dead cells. Subsequent gating was performed on single cells (FSC-A vs FSC-H) followed by gating on live cells as determined by exclusion of DAPI as a viability dye. The same gating strategy was used for Fig. 1A.
2. Surface staining on Hem14 WT1-CTLs for CD3 vs CD16 (top left), CD4 vs CD8 (top right), and CCR7 vs CD45RA (bottom right). Gating for CCR7 and CD45RA performed using matched PBMCs to differentiate positive vs negative populations (bottom left).
3. TAP-deficient, HLA-A*02:01-positive T2 cells were pulsed with increasing concentrations of WT1 peptide (amino acids 126-134) for 30min at 37deg Celsius, after which T2 cells were washed. T2 cells were then placed in co-culture with Hem14 WT1-CTLs (E:T 0.5) for 24hrs, at which point T2 cell viability was assessed. Data not shown on the graph is that T2 viability with no CTLs and no peptide (unpulsed T2 cells alone) had a mean viability of 77.3% (n=4).
4. PBMCs from donor Hem07 were sorted for CD8+ T cells (red bar) or underwent the ETC-generation process to make WT1-specific CTLs (grey bar). AML cell lines THP1 and OCIAML2 were treated with no CTLs (grey) or the above two CD8+ T cell populations. AML cell viability was assessed after 24hrs of co-culture CTLs at an E:T of 2.
5. AML cell viability after 24hrs of co-culture with Hem14 WT1-CTLs.
6. Gating strategy for assessing AML cell viability during co-culture with WT1-CTLs at 24hrs (as in Fig. 1D and Fig. S1E). Gating was first performed on all cells from a FSC-A vs SSC-A plot followed by selection for singlets based on FSC-A vs FSC-H. In this experiment, CTLs were labeled with cell trace violet (CTV) and thus gating was performed on the CTV-negative population (AML cells). The CTV-negative population was subsequently assessed for annexin V (FITC) vs propidium iodide (PI) and the viable population was defined as the double-negative population.

Results in C-E are shown as mean +/- standard deviation of n=3 (D) or n=4 (C, E) samples and are representative of 2 (C-E) independent experiments. ETC = endogenous T cell.

**Supplementary Figure 2**

(B)

(C)

(D)

**Fig. S2. Effect of venetoclax on MHC and TCR expression and effect of venetoclax on PBMC and CTL viability during AML cell co-culture**

1. PBMCs from donor Hem07 were placed in co-culture with WT1-CTLs from donor Hem14 (E:T 0.5) +/- venetoclax 100nM for 24hrs. Viability was assessed at 24hrs using annexin V / PI staining. PBMC viability was normalized to the untreated group (no VEN/no CTLs), which was set at 100% baseline viability. Results are shown as mean +/- standard deviation of n=4 samples. Asterisks are comparisons of each pair of dark grey vs light grey bars. * = *p* <0.05; ** = *p* <0.01 using unpaired *t* test.
2. AML cell lines were incubated with or without venetoclax 100nM for 24hrs in A221 media and subsequently assessed for surface expression of HLA-A2 (top row) and MHC class I (bottom row). Representative histograms are shown and median fluorescence intensities (MFI) are displayed comparing untreated to VEN-treated AML cells. Gating performed on live AML cells.
3. WT1-CTLs from 3 different donors were incubated with or without venetoclax 100nM for 24hrs in CTL media containing IL-15 (5ng/ml) and subsequently assessed for surface expression of TCRα/β. Gating performed on CD8+ cells. Representative histograms are shown and median fluorescence intensities (MFI) are displayed comparing untreated CTLs to VEN-treated CTLs
4. AML cells were treated for 24hrs with WT1 CTLs from donor Hem07 or donor Hem14 (E:T 0.5). Co-culture between CTLs and AML cells occurred without venetoclax (blue bars) or in the presence of venetoclax 100nM (red bars). Co-culture was with OCI-AML2 cells (left group), OCI-AML3 cells (middle group), or THP-1 cells (right group). WT1-CTL viability was assessed by annexin V / PI as described above in Fig. S1F, except that gating was performed on the CTL population rather than the AML population. CTL viability during co-culture in the absence of venetoclax was normalized for each pair to 100% to account for differences in donor CTL viability. Data is representative of two independent experiments. Statistical analyses are shown as mean +/- standard deviation of n=4 samples and ** indicates *p* < 0.01.

**Supplementary Figure 3**

(A)

(B)

**Fig. S3. Pre-treatment studies of VEN on AML cells or VEN on CTLs.**

1. AML cell lines were either pre-treated with or without VEN 100nM in A221 media for 24hrs. After VEN-exposure, AML cells were washed three times to remove VEN and then were placed in culture with or without WT1-CTLs (E:T 0.5) from donor Hem15 for an additional 24hrs. At the conclusion of 24hrs of co-culture (48hrs from experiment initiation), AML cell viability was assessed by annexin V / PI staining. Asterisks are for comparisons of CTL treatment alone vs VEN->CTL treatment; ** = *p* <0.01 using unpaired *t* test.
2. WT1-CTLs from donor Hem07 were either pre-treated with or without VEN (100nM and 200nM) in CTL media containing IL-15 (5ng/ml) for 24hrs. After VEN-exposure, CTLs were washed three times to remove VEN and then were placed in co-culture with AML cells (E:T 0.5) for an additional 24hrs. At the conclusion of 24hrs of co-culture (48hrs from experiment initiation), AML cell viability was assessed by annexin V / PI staining. Asterisks are for comparisons of co-culture with untreated CTLs vs VEN-treated CTLs. Statistical analyses were performed as a one-way ANOVA within each AML cell line between the 3 CTL treatment conditions (CTLs, VEN 100nM-treated CTLs, and VEN 200nM-treated CTLs) followed by post-hoc testing with an unpaired *t* test with a Bonferroni adjustment incorporated for significance (*p*/n) where n equals the number of paired comparisons per group (2 comparisons per group), yielding * = *p* <0.05/2 (*p* <0.025) and ** = *p* <0.01/2 (*p* <0.005).

Results are shown as mean +/- standard deviation of n=3 (A) or n=4 (B) samples.

**Supplementary Figure 4**

(A)

(B)

(C)

(D)

(E)

**BID**

**KO**

**SCR**


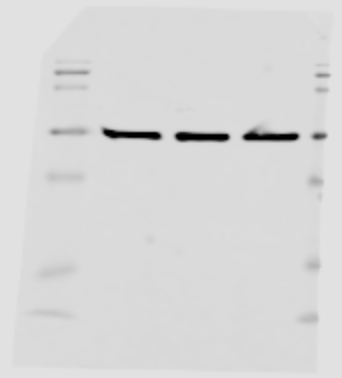


**⍺-tubulin**

**(50kDa)**

**50kDa -**

**25kDa -**


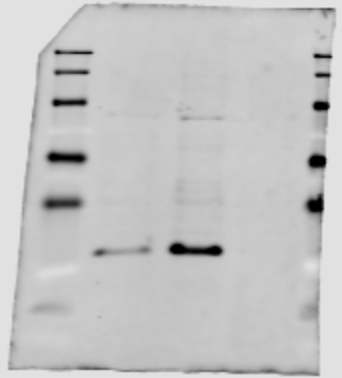


**BID**

**(22kDa)**

**15kDa -**

(F)

**Fig. S4. Venetoclax +/- WT1 CTLs on wild-type and KO AML cells and evaluation of death receptor blockade on CTL cytotoxicity of AML cells**

1. OCI-AML2 cells were co-cultured with WT1-CTLs (E:T 1) from donor Hem14 +/- venetoclax 100nM for 4hrs and 24hrs. Viability was assessed at the indicated timepoints using annexin V / PI staining. * = *p* <0.05; ** = *p* <0.01 using unpaired *t* test.
2. OCI-AML2 cells based on knockout status (negative control vs *BAX* KO vs *BAK* KO vs *BAX/BAK1* double KO) were treated with VEN (100nM) +/- WT1-CTLs (E:T 0.5) concurrently and AML cell viability was assessed at 24hrs using annexin V / PI staining. This is from the same experiment as shown in Figure 5C, however with single KO (*BAX* or *BAK*) data included in this figure. Statistical analyses were performed as a one-way ANOVA within each condition of 4 treatment groups (negative control, BAX KO, BAK KO, BAX/BAK KO) followed by post-hoc testing with an unpaired *t* test with a Bonferroni adjustment incorporated for significance (*p*/n) where n equals the number of paired comparisons per group (3 comparisons per group due to each KO compared to the negative control). Thus, * = *p* <0.05/3 (*p* <0.017); ** = *p* <0.01/3 (*p* <0.003) using unpaired *t* test in comparison to the black (negative control) bar within each condition.
3. OCI-AML3 cells were co-cultured with WT1-CTLs (E:T 0.5) from donor Hem14 for 4hrs +/- antibodies against TRAIL and Fas. For Fas blockade, OCI-AML3 cells were either treated with anti-Fas antibody (light blue) or isotype control (dark blue) and then placed into co-culture with WT1-CTLs. For TRAIL blockade, WT1-CTLs were either treated with anti-TRAIL antibody (light red) or isotype control (dark red) and then placed into co-culture with OCI-AML3 cells. Antibody remained in the media during the 4hrs of co-culture. Results are shown as the mean of n=2 samples and are representative of duplicate independent experiments.
4. OCI-AML3 (AML), Mel526 (melanoma), and Jurkat T cells were treated with soluble FasL (1ng/ml, 10ng/ml, 100ng/ml) or soluble TRAIL (0.5ng/ml, 5ng/ml, 50ng/ml) for 4hrs, at which point cell viability was assessed by annexin V / PI staining.
5. *BID* was knocked out from OCI-AML2 cells using CRISPR gRNA/Cas9 ribonucleoprotein nucleofection. Knockout efficacy was evaluated by western blot for Bid, with α-tubulin used as a loading control. Left column SCR = negative control OCI-AML2 (irrelevant scramble sgRNA); middle column = noncontributory cell lysate; right column = *BID* KO OCI-AML2. Molecular weight markers (15, 25, 50 kDa) are shown. Full western blot is available in the supplementary file. By densitometry, Bid was undetectable by western blot in Bid KO cells.
6. OCI-AML2 cells based on knockout status (negative control vs *BID* KO) were treated with VEN (100nM) +/- WT1-CTLs (E:T 0.5; CTL donor Hem07) concurrently and AML cell viability was assessed at 24hrs using annexin V / PI staining. * = *p* <0.05; ** = *p* <0.01 using unpaired *t* test. Results are representative of duplicate independent experiments.

Results are shown as mean +/- standard deviation of n=4 (A, D, F) or n=3 (B) samples.

**Supplementary Figure 5**

**Fig. S5. Characterization of primary AML samples**

Primary AML samples were stained for surface markers CD3, CD19, CD45, CD34, and CD38. Gating was performed on the live population of cells. Gating was performed using isotype control antibodies for each surface marker. Two representative samples are shown: patient 780 (top) and patient 382 (bottom).

**Supplementary Figure 6**

**Fig. S6. Cytotoxicity of WT1-CTLs against primary patient samples**

WT1-CTLs from Hem07 (left), Hem14 (middle), or Hem15 (right) donor were co-cultured with AML primary samples from 13 different patients (14 samples) in independent experiments. Cytotoxicity against the total sample (top row; characteristics of each sample outlined in Table 1) or, if applicable, against the CD34+/CD38- population (bottom) are shown. Details of individual values and E:T ratio used are outlined in Supplementary Table 1.

**Supplementary Figure 7**

**(A)**

**
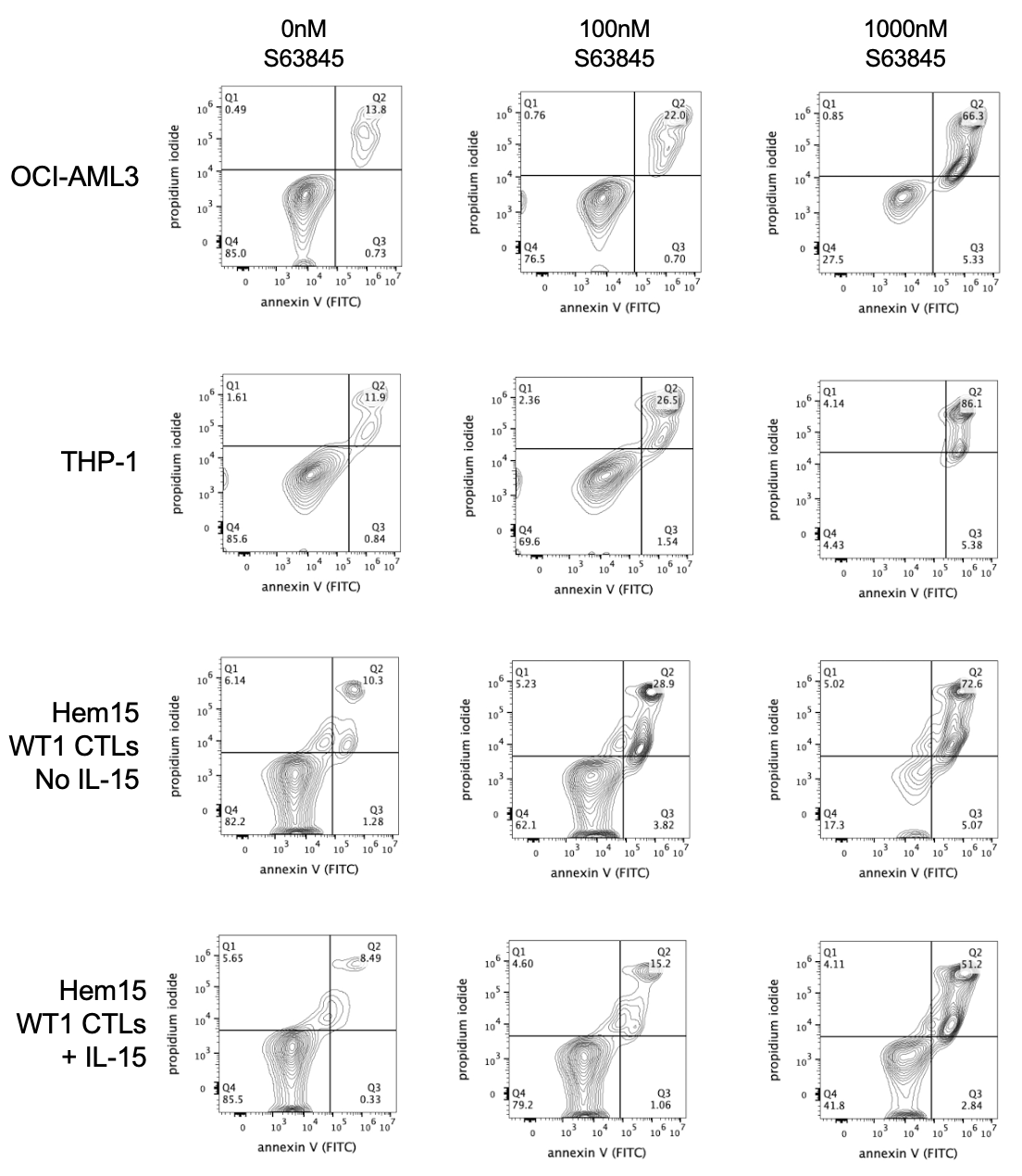
**

**(B)**

**Fig. S7. Role of intrinsic and extrinsic apoptotic pathways in S63845 + CTL treatment**

1. Representative flow plots of annexin V / PI staining are shown corresponding to data shown in Figure 7A/B. Left column shows samples with no S63845 exposure for 24hrs, middle column is after 24hrs of 100nM S63845 exposure, and right column is after 24hrs of 1000nM S63845 exposure. AML cell lines (OCI-AML3 and THP-1) are shown in the top two rows. The bottom two rows show viability of Hem15 WT1 CTLs without IL-15 included during S63845 exposure or with IL-15 (5ng/ml) included during S63845 exposure. Gating was performed on cells from a FSC-A vs SSC-A plot followed by selection for singlets based on FSC-A vs FSC-H. The single cell population was then assessed for annexin V (FITC) vs propidium iodide (PI) and the viable population was defined as the double-negative population.
2. THP-1 cells were placed in co-culture with WT1-CTLs (Hem14 donor, E:T 0.5) +/- S63845 (250nM). Prior to co-culture, CTLs were labeled with cell trace violet (CTV). At the indicated timepoints, CTV-negative (AML cells) were separated from CTV+ cells by FACS (5min sorting per sample) and CTV-negative cells were immediately placed in the indicated Caspase-Glo buffer for 45min prior to assessing luciferase activity. Equivalent numbers of CTV-negative cells were sorted for each condition at each timepoint.

Results in (B) are shown as mean +/- standard deviation of n=3 samples and are representative of duplicate independent experiments.

**Supplementary Table 1**

**
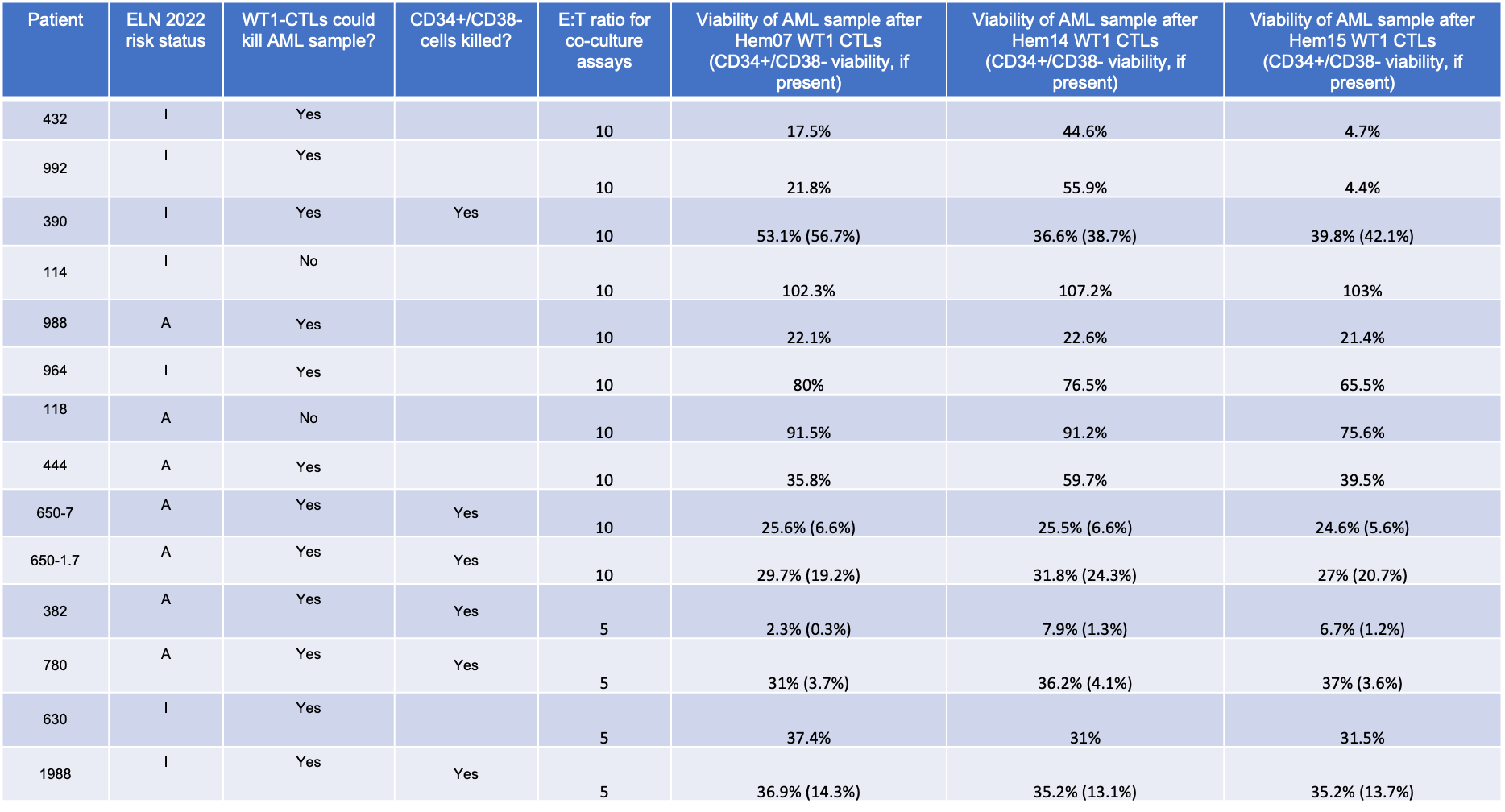
**

E:T = effector-to-target ratio

ELN = European LeukemiaNet; I = intermediate-risk; A = adverse-risk

Viability is normalized where 100% is viability for AML sample with no CTL treatment

Viability shown of total AML sample; in parenthesis, if applicable, CD34+/CD38- viability is also shown

E:T was 5-10 for all samples
