# Supplementary material for: BH3 mimetics augment cytotoxic T cell killing of acute myeloid leukemia via mitochondrial apoptotic mechanism
Source: Cell Death Discov. 2025 Mar 26;11:120. doi: 10.1038/s41420-025-02375-2 (PMC11947210; doi:10.1038/s41420-025-02375-2)
Supplement: Supplementary file 2 — Supplementary File With Full Western Blots [file 41420_2025_2375_MOESM2_ESM.docx]

**Supplementary File**

Western blot files for Figure 5B – full length uncropped western blot files

The membrane was scanned using an Odyssey CLx Imager (LI-COR Biosciences, Lincoln, NE) and normalized to the density of α-tubulin in the corresponding samples. Chameleon Duo Pre-stained Protein Ladder (LI-COR) was used as the ladder. Red is alpha-tubulin (DyLight 680). Green is anti-rabbit.

| **Antibody, species** | **Company** | **Catalog** |
| --- | --- | --- |
| Bax, rabbit | Cell Signaling Technology, Danvers, MA | 2772 |
| Bak, rabbit | Cell Signaling Technology | 12105 |
| Alpha-tubulin, mouse | Abcam, Cambridge, UK | ab7291 |
| anti-rabbit IgG, goat; DyLight 800 | Invitrogen/Thermo | SA5-10036 |
| anti-mouse IgG, goat; DyLight 680 | Invitrogen/Thermo | 35518 |

Columns (from left to right): ladder, SCR KO (scramble), BAX KO, BAK KO, BAX/BAK KO, ladder

Red bands only with exposure to see DyLight680 (excitation/emission max 676/705nm)


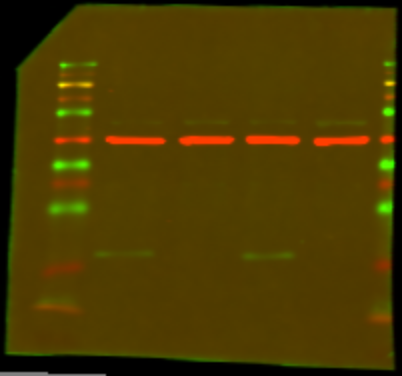

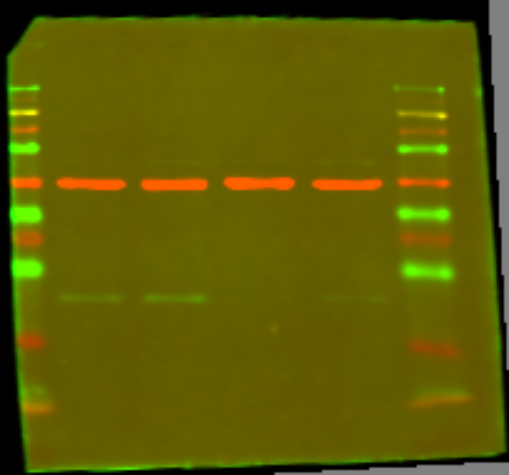
Green bands only with exposure to see DyLight800 (excitation/emission max 789/794nm)

BAX blot

BAK blot


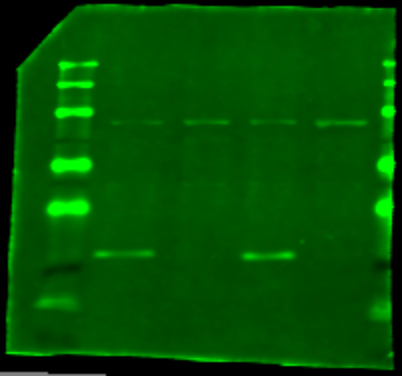

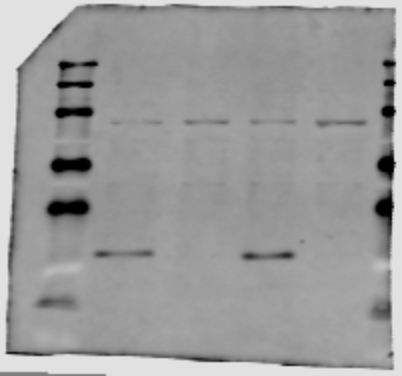

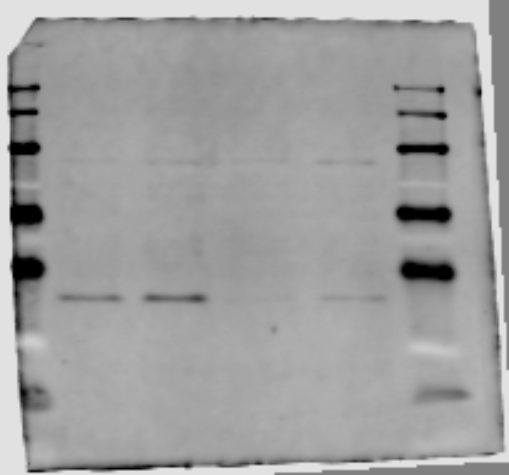

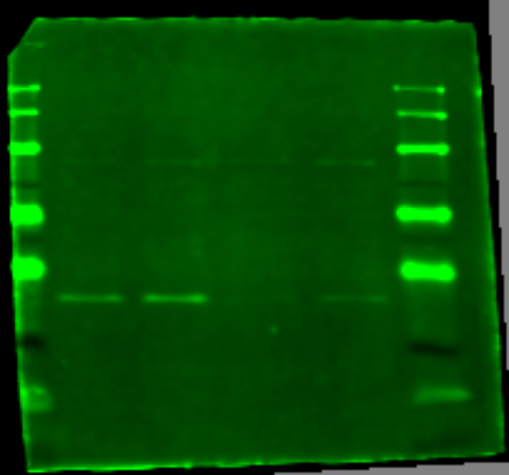

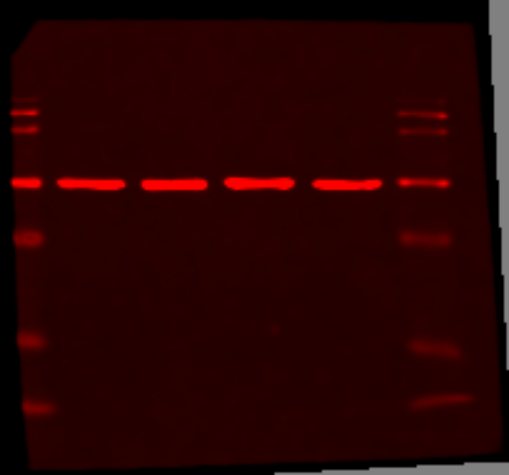

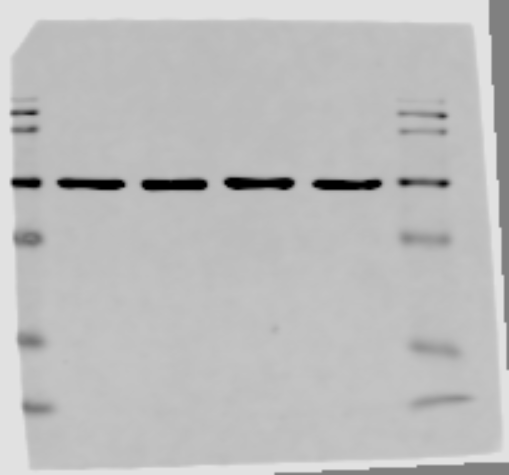

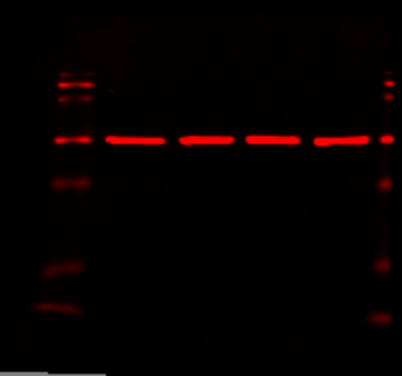

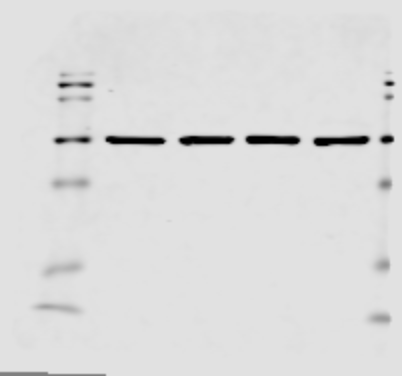


Green bands only

Green bands only

Red bands only

Red bands only

Western blot files for Supplementary Figure 4E – full length uncropped western blot files

The membrane was scanned using an Odyssey CLx Imager (LI-COR Biosciences, Lincoln, NE) and normalized to the density of α-tubulin in the corresponding samples. Chameleon Duo Pre-stained Protein Ladder (LI-COR) was used as the ladder. Red is alpha-tubulin (DyLight 680). Green is anti-rabbit.

| **Antibody, species** | **Company** | **Catalog** |
| --- | --- | --- |
| Bid, rabbit | Cell Signaling Technology | 2002 |
| Alpha-tubulin, mouse | Abcam, Cambridge, UK | ab7291 |
| anti-rabbit IgG, goat; DyLight 800 | Invitrogen/Thermo | SA5-10036 |
| anti-mouse IgG, goat; DyLight 680 | Invitrogen/Thermo | 35518 |

Columns (from left to right): ladder, SCR KO OCI-AML2 (scramble), additional/noncontributory cell lysate, BID KO OCI-AML2, ladder

Red bands only with exposure to see DyLight680 (excitation/emission max 676/705nm)

Green bands only with exposure to see DyLight800 (excitation/emission max 789/794nm)

BID blot


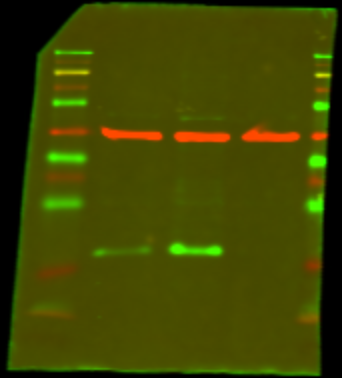


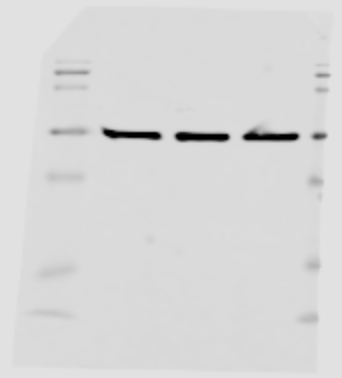
Red bands only


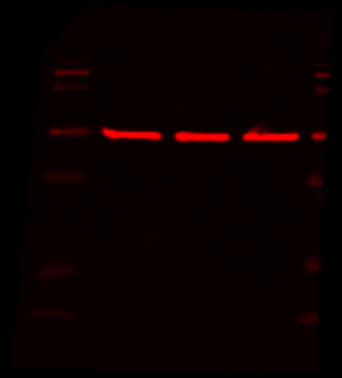


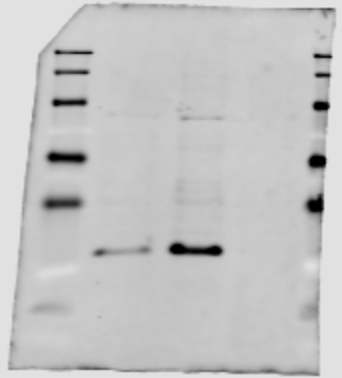

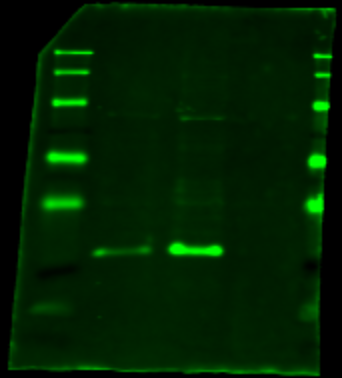
Green bands only
